# Does the implementation of clinical practice guidelines for low back and neck pain by physical therapists improve patient outcomes? A systematic review

**DOI:** 10.1186/s43058-022-00305-2

**Published:** 2022-06-03

**Authors:** Rebecca Fillipo, Katie Pruka, Marissa Carvalho, Maggie E. Horn, Jordan Moore, Benjamin Ramger, Derek Clewley

**Affiliations:** 1 Department of Physical Therapy and Occupational Therapy, Duke University Health System, Durham, North Carolina USA; 2 Department of Outpatient Rehabilitation, Duke University Health System, Durham, North Carolina USA; 3 Division of Doctor of Physical Therapy, Department of Orthopaedic Surgery, School of Medicine, Duke University, Durham, North Carolina USA; 4 Department of Population Health Sciences, School of Medicine, Duke University, Durham, North Carolina USA

**Keywords:** Low back pain, Neck pain, Implementation, Physical therapy

## Abstract

**Background:**

Physical therapy for neck and low back pain is highly variable despite the availability of clinical practice guidelines (CPG). This review aimed to determine the impact of CPG implementation on patient-level outcomes for spinal pain. Implementation strategies were also examined to determine prevalence and potential impact.

**Methods:**

Multiple databases were searched through April 2021 for studies assessing CPG implementation in physical therapy for neck and low back pain. Articles were screened for eligibility. The Modified Downs and Black checklist was utilized to determine study quality. Due to the heterogeneity between studies, a meta-analysis was not performed.

**Results:**

Twenty-one studies were included in this review. Implementation strategies were significantly varied between studies. Outcomes pertaining to healthcare utilization, pain, and physical functioning were assessed in relation to the implementation of CPGs. Multiple implementation strategies were identified, with *Managing Quality* as the most frequently utilized key implementation process. Findings indicate CPG implementation decreased healthcare utilization, but inconsistent results were found with physical functioning and pain outcomes.

**Conclusions:**

CPG implementation appears to have a beneficial effect on healthcare utilization outcomes, but may not impact pain and physical functioning outcomes. Effective CPG implementation strategies remain unknown, though utilizing implementation framework may improve outcomes. More research is needed to determine the most effective implementation strategies and effects on pain and physical function outcomes.

**Supplementary Information:**

The online version contains supplementary material available at 10.1186/s43058-022-00305-2.

Contributions to the literature

- This systematic review addresses gaps in the literature on how implementation of clinical practice guidelines, including the strategies utilized for implementation, impact outcomes of physical therapy care for patients with neck and low back pain.
- Guideline implementation appears to reduce health care utilization for patients with neck and low back pain.
- Implementation strategies for increasing adoption of clinical practice guidelines vary widely. This study recognizes opportunities for future research exploring the impact of implementation strategy on guideline adoption and patient outcomes including pain and physical functioning.

## Introduction

Low back pain (LBP) and neck pain are among the most common musculoskeletal complaints [1] and leading causes for patients to seek medical care [2]. Physical therapists frequently treat patients with low back and neck pain. However, it has been established that there is significant variability in the care provided to patients with low back and neck pain by physical therapists despite the existence of clinical practice guidelines (CPGs) to treat these conditions [3]. CPGs provide evidence-based recommendations to assist decision-making about health interventions [4]. These documents, developed by expert panels, are normally updated every 3 to 5 years or when the available evidence suggests a reformulation of the previous document is necessary [5]. CPGs are designed to support clinician decision-making through recommendations for evaluation and treatment, based on a synthesis of the best available evidence, to improve patient outcomes and guide physical therapists’ treatment planning and interventions. Numerous CPGs have been published on the management of LBP, both interdisciplinary and specific to physical therapy, though fewer have been published on the management of neck pain [6, 7]. Adherence to CPGs can decrease the use of ineffective treatments, decrease costs of treatment, and improve patient outcomes [8]. Thus the question remains: if adherence to CPGs improves care in patients with LBP and neck pain, why does treatment continue to be variable?

The authors propose that this variability in treatment may be due to the implementation strategy, or lack thereof, of CPGs. A recent systematic review examining musculoskeletal conditions reported that 54% of physical therapists chose treatments recommended by clinical practice guidelines, 43% chose treatments that were not recommended, and 81% chose treatments that have no recommendation [3]. Barriers to the use of CPGs by clinicians include lack of knowledge or awareness of CPGs, lack of access to recommendations, therapist beliefs, and patient expectations [9, 10]. These barriers can be overcome with effective, multifaceted implementation strategies targeting both individuals and healthcare systems to promote adherence to trustworthy CPGs [11]. Therefore, the purpose of this systematic review is to critically synthesize the literature regarding the implementation of CPGs for low back and neck pain in order to answer: (1) Does the implementation of guidelines by physical therapists improve patient outcomes for patients seeking care for low back and neck pain? and (2) Does the implementation strategy of CPGs impact outcomes?

## Methods

### Data sources and searches

The Preferred Reporting Items for Systematic Reviews and Meta-Analyses (PRISMA) guidelines were followed during the search and reporting phase of the research process [12]. The review was not registered, nor was a protocol prepared. A comprehensive literature search was performed in four electronic databases (MEDLINE, CINAHL, SCOPUS, and Embase) from inception to April 2020 and updated in May 2021. There exists significant overlap between SCOPUS and Web of Science, and therefore, we chose to only search SCOPUS [13]. OVID and PubMed exist as interfaces for the same database, MEDLINE; thus, only one interface needs to be searched in order to capture MEDLINE content. Cochrane was not searched as all published CPGs and randomized control trials are also indexed in MEDLINE. Grey literature was not searched and may be a limitation to this review. The literature search plan was developed and performed in collaboration with a Medical Librarian. The search was developed using keywords and subject headings, appropriate for each database, related to neck and/or back pain, as well as clinical practice guideline implementation. See Additional file 1.

### Selection criteria

Articles were eligible for study inclusion if they met the following criteria: (1) studies in which physical therapists or physiotherapists provided care for patients with neck and/or LBP, (2) patients were 18 years of age or older, (3) the physical therapists used published clinical practice guidelines for the management of neck or low back pain, (4) study designs included randomized clinical trials, observational cohort studies, and case reports published in English, and (5) included at least one outcome assessing pain, patient-reported physical functioning or disability, or healthcare utilization (HCU). We included all data pertaining to PT or other healthcare visits or associated costs and medication or procedure usage or costs associated with low back or neck pain under healthcare utilization.

### Study selection and data extraction

**Fig. 1:**
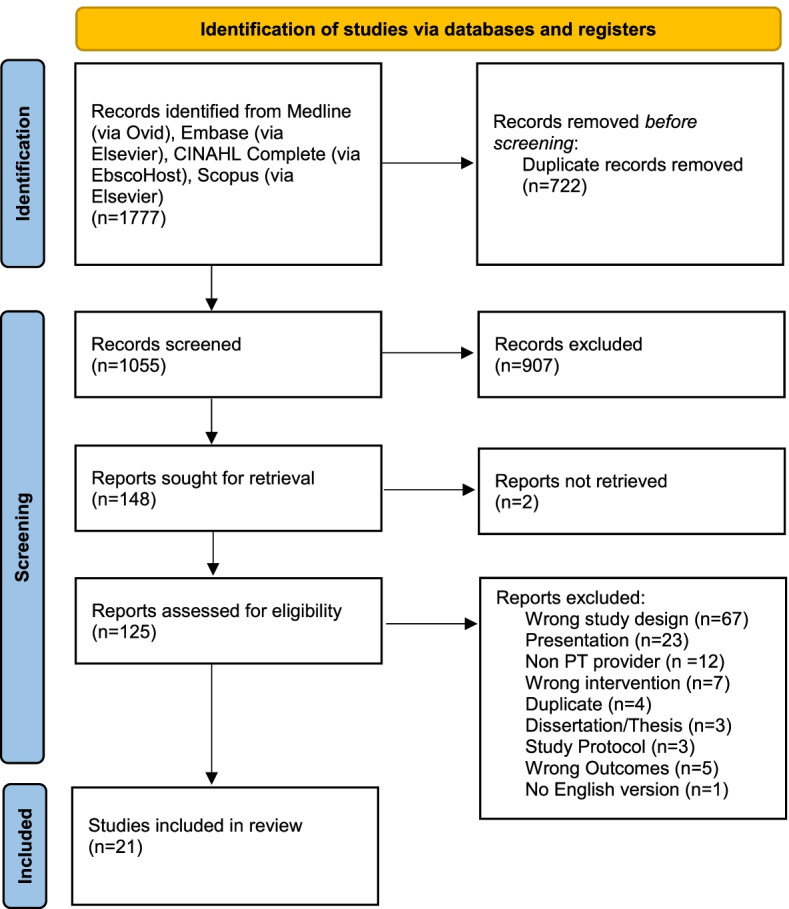
PRISMA diagram

Databases were systematically searched in April 2020 and updated in May 2021. All search results were imported into Covidence (Veritas Health Innovation, Melbourne, Australia). After duplicates were deleted, the titles and abstracts were screened by two reviewers (BR, RF), while one reviewer (KP) resolved any conflicts that arose between the two reviewers. References in systematic reviews were hand searched for additional articles. Full texts of the remaining articles were then screened by two reviewers (JM, RF), while one reviewer (MC) resolved any conflicts. Six reviewers (BR, JM, KP, MC, RF, DC) performed data extraction on the final studies included in the review. Each study had a primary data extractor and another author who checked for accuracy and completeness. A data extraction template was created. Each reviewer extracted this data from their assigned studies and input it into the template. The second assigned author checked for accuracy and completeness. See Fig. 1 for article selection.

### Data analysis

For this review, we descriptively reported our findings and did not perform a meta-analysis due to the lack of homogeneity of study designs and reported data across studies. We included all data reported in each study. First, we reported the study designs, study settings, sample sizes, and country where the studies were performed. Next, we examined the implementation processes and the implementation strategies for the guidelines that the studies reported. One challenge in implementation literature is that implementation strategies are not always clearly defined and there is often inconsistent language utilized. In an effort to clearly differentiate implementation strategies, we used the compilation by Powell et al. [14], which details six key implementation processes and 68 discrete, or individual, implementation strategies. The six key implementation processes represent larger overarching implementation processes and include planning, educating, restructuring, financing, managing quality, and attending to the policy context [14] and the 68 discrete implementation strategies are each classified under one of the key implementation processes. In this framework, planning includes strategies aimed at gathering information, selecting strategies, building buy-in, initiating leadership, and developing relationships. Educating strategies aim to inform individuals and include developing materials, educating, educating through peers, and informing and influencing stakeholders. Financing includes discrete strategies modifying incentives and facilitating financial support. Restructuring includes strategies addressing roles, sites, and systems. Managing quality includes putting strategies and systems in place to evaluate and improve quality. Attending to the policy context includes addressing requirements, laws, and standards. Using this compilation by Powell et al. [14] allowed for assessment of implementation strategies at both a broad level using the key processes and a focused level using the specific strategies. One reviewer (MC) retroactively assigned the discrete implementation strategies and key implementation processes to each study based on reviewer interpretation of the implementation process described in each and one reviewer (RF) checked for accuracy and completeness.

Lastly, we examined primary and secondary clinical outcomes pertaining to HCU, pain, and patient-reported physical function and disability. HCU included healthcare visits, healthcare costs, physical therapy visits and physical therapy costs, procedural interventions, medication utilization, and imaging. Physical function and disability were assessed using patient-reported outcomes including Roland-Morris Disability questions, Oswestry Disability Index, Patient-Reported Outcomes Measurement Information System (PROMIS), or other measures as indicated by the study. Studies were organized according to the outcomes assessed.

### Quality assessment

Risk of bias was assessed using the Downs and Black checklist for internal consistency among the different study types included, without considering the power analysis. This has been shown to be a reliable and valid tool for measuring the methodological quality of randomized and nonrandomized studies [15]. Two reviewers (RF, BR) evaluated each study independently. A third reviewer (DC) settled any discrepancies between the primary reviewers.

## Results

### Study characteristics

#### Study design

Twenty-one studies were included for final review. The characteristics of the final included studies are presented in Table 1. All twenty-one studies were published between 2005 and 2021. Of the twenty-one studies retained for final review, seven were retrospective cohort studies [16–22], seven were randomized controlled trials [23–29], six were prospective, longitudinal cohort studies [30–35], and one was a case report [36].

**Table 1:** Study characteristics

Author, year	Sample size	Design	Clinic setting; country of origin	Key implementation process	Implementation strategy	Risk of bias (Downs & Black)
**Low back pain**						
Bekkering, 2005 [29]	500 Patients 113 PTs	Cluster RCT	Outpatient Clinic: Private Practice; The Netherlands	Planning; Educating; Managing Quality	Tailor strategies to overcome barriers and honor preferences; distribute education materials; conduct educational meetings; make training dynamic; model and simulate change; audit and provide feedback; remind clinicians	23
Childs, 2015 [22]	753,540 Patient charts 112,723 (16%) Patients utilizing physical therapy	Retrospective Cohort	Military Treatment Facilities and Clinics Reimbursed by TriCare; Global: MHS	Managing Quality	Use data warehousing techniques; use data experts; audit and provide feedback	17
Feuerstein, 2006 [33]	15,789 Patients	Longitudinal Panel Study and Cross Sectional	Military Healthcare System Outpatient Clinics; Global: MHS	Planning; Educating; Managing Quality	Conduct local needs assessment; identify and prepare champions; use advisory boards and work groups; develop a formal implementation blueprint; develop effective educational materials; distribute educational materials; purposely re-examine the implementation; audit and provide feedback; use data warehousing techniques; use data experts	20
Fritz, 2012 [16]	32,070 Patients 2234 Patients utilizing physical therapy	Retrospective Cohort	Outpatient Clinics: Private, Non-profit; United States: Utah	Managing Quality	Use data warehouse techniques; use data experts; audit and provide feedback	20
Hoeijenbos, 2005 [25]	500 Patients 113 Physiotherapists	Cluster RCT	Outpatient Clinic: Private Practice; The Netherlands	Educating; Managing Quality	Distribute education materials; conduct educational meetings; make training dynamic; develop effective educational materials; audit and provide feedback; remind clinicians	19
Karlen, 2015 [36]	47,755 Patient episodes 32 Outpatient clinics	Case Report	Outpatient Clinics; United States	Planning; Educating; Restructuring; Managing Quality	Conduct local needs assessment; assess for readiness and identify barriers; tailor strategies to overcome barriers and honor preferences; conduct local consensus discussions; involve executive boards; identify and prepare champions; recruit designate and train for leadership; mandate change; develop effective educational materials; distribute educational materials; conduct educational meetings; conduct ongoing training; make training dynamic; revision of professional roles; facilitate relay of clinical data to providers; develop and organize quality monitoring systems; purposely re-examine the intervention; audit and provide feedback; use advisory boards and work groups; capture and share local knowledge; organize clinician implementation team meetings	12
Kongsted, 2019 [32]	250 Patients 31 Clinicians	Longitudinal Cohort, feasibility study	Outpatient Clinics; Denmark	Planning; Educating; Managing Quality	Develop academic partnerships; work with educational institutions; conduct educational meetings; make training interactive; distribute educational materials; develop and organize quality monitoring systems; audit and provide feedback; purposely re-examine the implementation; use data warehousing techniques	16
Lemieux, 2021 [30]	78 Patients 35 Clinicians	Longitudinal Cohort, feasibility study	Outpatient Clinics; Canada	Educating ; Managing Quality; Financing	Conduct educational meetings; develop effective education materials; make training dynamic; distribute educational materials; purposely re-examine the implementation; use data warehousing techniques; use other payment schemes	16
Magel, 2018 [34]	400 Patients	Prospective Observational Cohort	Outpatient Clinics; United States	Planning; Managing Quality	Conduct local needs assessment; assess for readiness and identify barriers; tailor strategies to overcome barriers and honor preferences; use advisory boards and workgroups; audit and provide feedback, purposely re-examine the implementation; remind clinicians; intervene with patients/consumers to enhance uptake and adherence	20
Rutten, 2010 [35]	145 Patients 61 PTs	Prospective Observational Cohort	Outpatient Clinics: Private Practice; The Netherlands	Managing Quality	Use data warehousing techniques; use data experts; audit and provide feedback; develop tools for quality monitoring	18
Sharma, 2019 [26]	40 Patients	Two-arm, parallel, assessor-blinded, feasibility RCT	Rehabilitation Hospital; Nepal	Planning; Educating	Conduct local needs assessment, Develop effective educational materials, involve patients/consumers and family members, Distribute educational material	22
Schroder, 2021 [23]	500 Patients 123 PTs	Cluster RCT	Outpatient Clinics: Public ; Sweden	Planning; Educating; Managing Quality	Conduct local needs assessment; Tailor strategies to overcome barriers and honor preferences; Develop a formal implementation blueprint; Identify and prepare champions; Develop effective education materials; Distribute education materials; Conduct educational meetings; Conduct ongoing training; Use advisory boards and workgroups; Obtain and use patient/consumer and family feedback; Purposely re-examine the implementation	20
Swinkels, 2005 [21]	1254 Patient charts 90 Therapists from 40 practices	Retrospective Cohort	Outpatient Clinics: Private Practice; The Netherlands	Educating; Managing Quality	Distribute education materials; use data warehousing techniques; use data experts; audit and provide feedback	17
**Acute low back pain**						
Fritz, 2007 [17]	1190 Patient charts	Retrospective Cohort	Outpatient Clinics: Private, Non-profit; United States: Utah	Managing Quality	Use data warehousing techniques; use data experts; audit and provide feedback	18
Fritz, 2008 [18]	471 Patients	Retrospective Cohort	Outpatient physical therapy clinics in the Rehabilitation Agency of Intermountain Healthcare (IHC) system; United States: Utah	Educating; Managing Quality	Distribute educational materials; use data warehousing techniques; use data experts; audit and provide feedback	14
Owens, 2019 [20]	105 Patients	Retrospective Cohort	Outpatient: Workers Compensation; United States	Managing Quality	Use data warehousing techniques; use data experts; audit and provide feedback	19
**Chronic low back pain**						
Van der Roer, 2008 [24]	120 Patients	RCT	Outpatient Clinics; The Netherlands	Managing Quality	Use data experts; audit and provide feedback	16
**Low back pain with radicular symptoms**						
Fleuren, 2010 [31]	723 Patients 360 General practitioners, 550 physiotherapists, 9 neurologists, 18 radiologists	Longitudinal Cohort	Outpatient Rehabilitation Clinics; The Netherlands	Planning; Educating; Managing Quality	Conduct local needs assessment; assess readiness and identify barriers; tailor strategies to overcome barriers and honor preferences; conduct local consensus discussions; recruit, designate, and train for leadership; develop effective educational materials; distribute educational materials; conduct educational meetings; conduct ongoing training; provide ongoing consultation; use mass media; audit and provide feedback; remind clinicians; purposely re-examine the implementation; use an improvement/implementation advisor; organize clinician implementation team meetings	16
Hahne, 2017 [27]	54 Patients	Subgroup analysis of multicenter RCT	Outpatient Clinics; Australia	Educating; Managing Quality	Conduct educational meetings; develop effective education materials; distribute education materials; provide ongoing consultation; audit and provide feedback	22
**Neck pain**						
Cote, 2019 [28]	340 Participants	Pragmatic RCT	Multidisciplinary Rehabilitation Clinics; Canada	Financing; Educating	Place on fee for service lists/formularies; fund and contract for the clinical innovation; conduct educational meetings; distribute education materials	26
Horn, 2016 [19]	298 Patients	Retrospective Cohort	Outpatient Clinics; United States	Managing Quality	Use data warehousing techniques; use data experts; audit and provide feedback	19

#### Risk of bias

The majority of the studies included were of moderate risk of bias, with one study [36] at high risk of bias. Only three studies had a low risk of bias [26, 27, 29], scoring at least 22 points on the Downs and Black checklist.

#### Study population

Nineteen studies assessed the impact of guideline implementation in patients with LBP [16–18, 20–27, 29–36] and two studies assessed guideline implementation in patients with neck pain [19, 28]. Of the studies that assessed guideline implementation and LBP, two specifically included only back-related leg pain [27, 31] and three included only acute LBP [17, 18, 20]. The remaining studies examined patients with non-specific LBP. Of the two studies analyzing guideline implementation and neck pain, one study specifically assessed application in patients with acute whiplash-associated disorders (WAD) [28]. Sample sizes in the studies examined ranged from 40 to 753,540 patients, with higher sample sizes found in the retrospective cohort studies.

#### Study setting

Seven studies were conducted in the USA [16–20, 34, 36], six studies were conducted in the Netherlands [21, 24, 25, 29, 31, 35], and two studies were conducted in Canada [28, 30]. The remainder of the studies were conducted in Nepal [26], Denmark [32], Sweden [23], and Australia [27]. Two did not report a country as they were conducted in global military health systems [16, 33]. Five studies were conducted in private practice clinics [16, 18, 21, 29, 35], two studies were conducted in military treatment centers [16, 33], and one study was conducted based on a review of workers compensation [20]. The remainder of the studies were conducted in the outpatient setting.

### Implementation strategies

The majority of studies included strategies from multiple key implementation processes and the classifications can be found in Table 1. Seven studies [16, 17, 19, 20, 22, 24, 35] used strategies within only one key implementation process, all of which used strategies within *Managing Quality*. *Managing Quality* was the most prevalent key implementation process, with nineteen studies [16–25, 27, 29–36] using this approach. Thirteen studies [18, 21, 23, 25–33, 36] used strategies within the *Educating* key implementation process, eight studies [23, 26, 29, 31–34, 36] used strategies within the *Planning* key implementation process, two studies [28, 30] used strategies within the *Financing* key implementation process, and one study [36] used strategies within the *Restructuring* key implementation process. No studies used strategies within the *Attending to Policy Context* key implementation process.

Four studies [23, 25, 29, 34] utilized published implementation frameworks. One [34] followed the RE-AIM framework. Two articles [30, 32] used COM-B from the Behavior Change Wheel. One [23] used COM-B and the Theoretical Domains Framework (TDF). Two additional studies [33, 36] used psychological and educational theories to guide implementation interventions. One [31] reported using an implementation specialist, but did not disclose additional details.

### Health care utilization

#### Cost

Eighteen studies [16–28, 31, 33–36] assessed HCU as an outcome. Eight studies [16, 18–20, 22, 24, 25, 33] assessed total healthcare costs, and six studies [17–19, 24, 25, 36] assessed PT episodes of care costs only. All studies assessing costs relative to guideline adherence found decreased costs for guideline adherent care except one [19], which found no difference between groups for non-PT healthcare costs. Hoeijenbos et al. [25] found no significant differences in cost between active and standard guideline implementation except for a difference in healthcare costs at the 6-week mark. Standard implementation involved passive dissemination of guidelines, whereas active implementation involved two training session in addition to the standard implementation [25]. One study [24] found significantly lower costs for patients receiving guideline-based care compared to an intensive group training protocol (Table 2).

**Table 2:** Reported healthcare and PT costs by study

Author, year	Healthcare costs	PT costs
**Low back pain**		
Childs, 2015 [22]	Adherent $2426.88 (SE 30.04) Nonadherent $2733.57 (SE 26.92) Difference $306.69 (95% CI 227.63 to 385.75) Early $1828.24 (SE 15.28) Delayed $3030.53 (26.64) Difference $1202.29 (95% CI 1142.09 to 1262.49)	
Feuerstein, 2006 [33]	Guideline adherent $222.40 Nonadherent $712.60 **(*p*< .0001)**, B = −230.10 (95% CI −264.1 to 195.9)	
Fritz, 2012 [16]	Adherent: LBP-related costs were an average $1374.30 lower favoring adherent care vs nonadherent 95% CI 202.28 to 2546.31	
Hoeijenbos, 2005 [25]	Mean Direct Medical Costs: Same pattern in the intervention and control group over time: a rapid decrease in the first 12 weeks and after 6 months the healthcare utilization stabilized. Peak consumption 6 weeks Baseline total direct medical cost: Intervention € 92, median € 72, (SD 62); Control € 89 median € 71, (SD 69) 6 weeks total direct medical cost: Intervention € 125 median € 111, (SD 91) Control € 145 median € 141, (SD 95) ***P*=.026** 12 weeks total direct medical cost: Intervention € 58 median € 20, (SD 91), Control € 77 median € 25, (SD 107) *P* = .051 26 weeks total direct medical cost: Intervention € 33 median € 0, (SD 98), Control € 35, median € 0, (SD 99) *P* =.818 52 weeks total direct medical cost: Intervention € 24 median € 0, (SD 68), Control € 30, median € 0, (SD 109) *P* =.477 Increase in costs at 6 weeks and decrease at 12 and 26 weeks were significant within both groups (***P*< 0.000**) Mean annual direct costs: Intervention € 374 (SD 427) Control € 449 (SD 572) Mean annual productivity costs: Intervention € 4838 (SD 9572) Control € 4035 (SD 8962) Costs per visit: General practitioner (one visit) € 18.37 Company doctor (one visit) € 18.37 Medical specialist (one visit) € 45.22 1 day in hospital € 261.23 Alpha help per hour € 9.44 Cost-effectiveness of intervention was not calculated due to lack of significant differences, likely extended implementation strategy increases costs	Mean direct medical costs for physiotherapists the previous 6 weeks: Baseline: Intervention € 54, median € 40; Control € 52, median € 40 6 weeks: Intervention € 106, median; 101 Control € 125 median € 121 12 weeks: Intervention € 51 median € 0.00; Control € 61 median € 0 26 weeks: Intervention € 18 median; € 0 Control € 22 median € 0 52 weeks: Intervention € 15 median; € 0 Control € 19 median € 0 Physiotherapist costs include physiotherapist, manual therapist and Mensendieck or Cesar therapist Costs per visit: Physiotherapist (one visit) € 20.10 Physical therapist (one visit) € 19.70 Manual therapist € 30.80 Physiotherapist per hour € 26.42
Karlen, 2015 [36]		Physical Therapy Charges: 2010: Adherent $773, Nonadherent $806 2011: Adherent $815, Nonadherent $861 2012: Adherent $847, Nonadherent $863 2013: Adherent $906, Nonadherent $969 2014: Adherent $896, Nonadherent $976 Increase in charges per LBP episode was 40% lower than the observed rate of inflation for individual units of PT
**Acute low back pain**		
Fritz, 2007 [17]		Adherent $845.57 (SD $449.14) Nonadherent $884.91 (SD $523.37), ***P*< .001**
Fritz, 2008 [18]	Additional charges for healthcare associated with LBP (1 year after completion of PT): 296 patients (62.8%) Cost: Mean charges: Adherent $1692 (SD $7683) Nonadherent $2829 (SD $21,728, ***P*< .05**) Receiving adherent physical therapy care was associated with a reduced likelihood of incurring high charges for subsequent healthcare. aOR = 0.51 (95% CI 0.31 to 0.87). Mean overall charges for care (charges for physical therapy+charges for subsequent healthcare): Adherent: $2255 (SD $7665) Nonadherent: $3559 (SD $21,720, ***P*< .05**) Adherent physical therapy care: reduced likelihood of incurring high overall charges. aOR = 0.44; (95% CI 0.26 to 0.75).	Adherent $562 (SD 269) Nonadherent $729 (SD 345) ***P*<.05**
Owens, 2019 [20]	Medical cost (median): $770, range 0–24,327 Total cost (median): $987, range 124–63,992 Each unit increase in ACOEM +1/−1 compliance: average $352.90 reduction in medical costs (*P* = .075) and $586.20 reduction in total costs (*P* = .22) Expensive outliers were consistent with lower scores, suggesting lower compliance results in higher costs Statistically significant relationship (***P*= .0097**) between decreasing claim's medical costs and increasing compliance with the ACOEM guidelines when log-transformed to better account for skewed cost distribution and outliers	
**Chronic low back pain**		
Van der Roer, 2008 [24]	Direct health care costs: Protocol € 1003 (SD 595), Guideline € 527 (SD 447), Mean difference € 475, (95% CI 211 to 681) Direct non-health care costs: Protocol € 82 (SD 233), Guideline € 197 (SD 463) Mean Difference € −115, (95% CI −220 to 27) Functional Status (RDQ): Cost Difference € 233, (95% CI −2185 to 2764) Effect Difference 0.06, (95% CI −2.22 to 2.34) Incremental Cost-Effectiveness Ratios (ICER) 16,349 Pain Intensity (PI-NRS): Cost Difference € 233, (95% CI −2185 to 2764) Effect Difference −1.02, (95% CI −2.14 to 0.09) ICER −175 Perceived Recovery (GPE): Cost Difference € 233, (95% CI −2185 to 2764) Effect Difference 13%; OR = 1.71, (95% CI 0.67 to 4.38) ICER 1720 QALY_NL_ (EQ-5D): Cost Difference € 233, (95% CI −2185 to 2764) Effect Difference 0.03, (95% CI −0.06 to 0.12) ICER 5141	Protocol € 779 (SD = 0) Guideline € 312 (SD = 191) Mean Difference € 467, (95% CI 298 to 646)
**Neck pain**		
Horn, 2016 [19]	No significant difference in costs to non-PT health providers. *e*^*B*^ = 0.79, 95% CI 0.26 to 2.24; *P* = .68	Adherent care: 22% lower charges for PT. Mean difference US$ 172.55; *e*^*B*^ = 0.78, 95% CI 0.69 to 0.89; ***P*< .001**

Bolded indicates statistical significance

*PT* physical therapy, *SE* standard error, *SD* standard deviation, *aOR* adjusted odds ratio, *RDQ* Roland Morris Disability Questionnaire, *EQ-5D* EuroQol-5D, *ICER* incremental cost-effectiveness ratio

#### Visits

Eight studies [17, 18, 23, 24, 26, 27, 30, 33] assessed the total number of healthcare visits, fifteen studies [16–19, 21–28, 34–36] assessed the number of PT visits, and four studies [17, 18, 23, 34] assessed the duration of PT care. One study [16] identified predictors of PT utilization for patients with low back pain, including higher index visit copayment, not receiving long-term disability, greater number of diagnosis codes at index visit, and not having comorbid neck/thoracic pain. Of the six studies assessing visits relative to guideline adherence, four [17–19, 35] found significantly decreased visits for guideline adherent care. The other two [22, 36] found similar trends but did not perform a statistical analysis. Outcomes pertaining to visits can be found in Table 3.

**Table 3:** Reported healthcare and PT visits by study

Author, year	Healthcare visits	PT visits / duration
**Low back pain**		
Childs, 2015 [22]		Mean PT visits: Adherent 6.2 (SD 7.6) Nonadherent 15.0 (SD 17.2) Early 7.3 (SD 12.9) Delayed 6.8 (11.0)
Fritz, 2012 [16]		PT utilization: 7.0% in first 90 days Visits: mean 6.4 (SD 5.1), 14.2% received only one visit 53.1% received early physical therapy, 46.9% received delayed Median time to PT: 14 days (IQR 6–33) Predictors of PT Utilization: higher index visit copayment; aOR =1.02; ***P*= 0.022** not receiving long-term disability: aOR = 0.21; ***P*=.04** greater number of diagnosis codes at index visit: aOR =1.04; ***P*<.001** not having comorbid neck/ thoracic pain: aOR = .76; ***P*<.001** Midwest as the reference, utilization in: Northeast, aOR = 1.59; ***P*<.001** West aOR =1.61, ***P*<.001** not living in the South: aOR = 0.82; ***P*=.004** Early PT: LBP-related costs were $2736.23 lower (95% CI 1810.67 to 3661.78) decreased likelihood of advanced imaging: OR = 0.34, (95% CI 0.29 to 0.41) additional physician visits: OR = 0.26, (95% CI 0.21 to 0.32)
Hoeijenbos, 2005 [25]	General practitioner utilization. all: Baseline 94% 6 weeks 25% 12 weeks 10% 4 patients were hospitalized during the 1-year follow-up for an average of 1.5 days Utilization in 6 weeks GP Contact: Baseline: Intervention 93.8%, mean 1.4, median 1; Control 94.6%, mean 1.6, median 1 6 weeks: Intervention **23.6%, mean 0.42, median 0.0**; Control: **25.7%, mean 0.45, median 0.0** 12 weeks: Intervention **7.2%, mean 0.13, median 0.0**; Control: **14.5%, mean 0.32, median 0.0** 26 weeks: Intervention 10.2%, mean 0.17, median 0.0; Control: 11.5%, mean 0.27, median 0.0 52 weeks: Intervention 7.5%, mean 0.13, median 0.0; Control: **5.6%**, mean 0.085, median 0.0 Hospitalization: Baseline: Intervention 0.0% Control 0.0% 6 weeks: Intervention 0.0% Control 0.0% 12 weeks: Intervention 0.5% control 0.5% 26 weeks: Intervention 0.5%, Control 0.0% 52 weeks: Intervention 0.0% Control 0.5% Bolded denoted significant difference from previous measure	Utilization in 6 weeks: Physiotherapist Contact: Baseline: Intervention 89.5%, mean 2.0, median 1; Control 86.3%, mean 2.1, median 2.0 6 weeks: Intervention **75.3%, mean 4.17, median 4;** Control: 80.7%, **mean 5.43, median 5.0** 12 weeks: Intervention **40.8%, mean 2.01, median 0.0;** Control: 48.2%, **mean 2.74, median 0.0** 26 weeks: Intervention **11.2%, mean 0.62, median 0.0;** Control: 16.2%, **mean 0.90, median 0.0** 52 weeks: Intervention 7.5%, mean 0.42, median 0.0; Control: **8.0%**, mean 0.42, median 0.0 Physical Therapist Contact: Baseline: Intervention 2.9%, mean 0.1, median 0.0; Control 2.1%, mean 0.058, median 0.0 6 weeks: Intervention 3.0%, mean 0.1, median 0.0; Control 3.1%, mean 0.16, median 0.0 12 weeks: Intervention 3.6%, mean 0.14, median 0.0; Control 0.5%, mean 0.09, median 0.0 26 weeks: Intervention 1.4%, mean 0.12, median 0.0; Control 1.4%, mean 0.07, median 0.0 52 weeks: Intervention 4.2%, mean 0.22, median 0.0; Control 4.2%, mean 0.19, median 0.0 Manual Therapist Contract: Baseline: Intervention 20.9%, mean 0.52, median 0.0; Control 12.4%, mean 0.27, median 0.0 6 weeks: Intervention **15.3%, mean 0.67, median 0.0;** Control **9.2%, mean 0.41, median 0.0** 12 weeks: Intervention **7.6%, mean 0.25, median 0.0**; Control **6.3%, mean 0.18, median0.0** 26 weeks: Intervention **2.8%, mean 0.11, median 0.0;** Control: **1.8%, mean 0.07, median 0.0** 52 weeks: Intervention 3.7%, mean 0.08, median 0.0; Control: 4.2%, mean 0.073, median 0.0 Bolded denoted significant difference from previous measure
Karlen, 2015 [36]		Mean number of visits: 2010: Adherent 5.1; Nonadherent 6.2 2011: Adherent 5.3; Nonadherent 6.4 2012: Adherent4.9; Nonadherent 5.7 2013: Adherent 4.8; Nonadherent 5.8 2014: Adherent 4.5; Nonadherent 5.7 Overall mean number of visits: 2010: 6.7 visits 2014: 5.4 visits
Magel, 2018 [34]	Spine surgeon visit: All patients 25 (6.3%) Participants 3 (2.4%) Nonparticipants 22 (8.0%) Refused 8 (7.9%) Not offered 14 (8.0%) Attended physiatry within 6 months of index visit: Al Patients 327 (81.8%) Participants (41.1%) Nonparticipants 276 (100%) Refused 101 (100%) Not offered 175 (100%) Participants were less likely to have visits to spinal surgeon over the 6 month follow period compared to Nonparticipants (***P*< .05)**	Days to schedule initial PT visit (median days, IQR): PT via RapidAccess 2 (1, 5) PT following physiatrist visit 36 (12.5, 77.5) Mean number of PT visits: PT via Rapid Access 4.3 (SD 3.6) PT following physiatrist visit 4.8 (SD 4.4) PT via RapidAccess 25 (20.2%) attended 1 visit PT following physiatrist visit 18 (18.8%) attended 1 visit Mean duration of PT care: PT via Rapid Access 42 (SD 15; days 92) PT following physiatrist visit 49 (SD 24; days 102) ***P*= .045**
Rutten, 2010 [35]		Mean number of treatment sessions 6.7 (SD = 3.2) Association between % guideline adherence and number of sessions: B = −0.09, Beta= −0.27, ***P*= .005** Association between % guideline adherence for individual steps and number of sessions: Treatment plan: Beta = −0.02 ***P*= .05** Evaluation: Beta= −0.03 ***P*= .01** Treatment: beta= −0.03 ***P*= .00** Correlation of % Adherence and Difference in Scores for No. of Sessions: Acute LBP (<6 weeks) −0.30, ***P*< .05** Subacute LBP (6–12 weeks) −0.28 Chronic LBP (>12 weeks) −0.37, ***P*< .05**
Sharma, 2019 [26]		Regular physiotherapy at the center: Pain Education 4 (21%) Control 5 (26%) *P* = 0.719
Schroder, 2021 [23]		Number of PT treatment sessions Intervention 4.6 (SD 3.8) Control 3.1 (SD 2.7) Duration PT intervention period: Intervention 63 (SD 61) Control 59 (SD 84)
Swinkels, 2005 [21]		Number of treatment sessions: Acute: median 8.0 (IQR = 4.5–12) Chronic: median 9.0 (IQR = 6–14)
**Acute low back pain**		
Fritz, 2007 [17]		Visits (median, range): Adherent care 5 (3–21), Nonadherent care 6 (3–35), ***P*= .02** Duration of Episode of PT Care (median, range): Adherent 20 (10–124) Nonadherent 26 (10–250), ***P*< .001**
Fritz, 2008 [18]	Rate of additional healthcare utilization: Adherent 55.3%, Nonadherent 65.8%, ***P*< 0.05**	Visits: Adherent group 4.6 (SD 2.0), Nonadherent group 5.9 (SD 2.2); ***P*= .02** Duration of care (days): Adherent group 25.4 (SD 16.2), Nonadherent group 29.7 (SD 20.6); ***P*< .001**
**Chronic low back pain**		
Van der Roer, 2008 [24]	No significant difference between groups General practitioner (consultations): Protocol 1.5 (SD 2.8), 45.5% Guideline 1.4 (SD 1.9), 55.3% Outpatient visit (no.): Protocol 0.6 (SD 2.4), 16.4% Guideline 0 (SD 0), 0% Hospitalizations (days): Protocol 0.1 (SD 0.4), 5.% Guideline 0 (SD 0), 0%	Physical Therapy (treatment sessions): Protocol 1.1 (SD = 4.1), 9.1% Guideline 2.1 (SD = 5.7) 17.0%
**Low back pain with radicular symptoms**		
Fleuren, 2010 [31]	Unnecessary Early Referrals: Pretest: All 27 (15%) First Post-test: **All 19 (9%); aOR = 0.52 (95% CI 0.28 to 0.96)** **Fast track 7 (6%); aOR = 0.36 (95% CI 0.15 to 0.86)** Standard 12 (11%); aOR = 0.69 (95% CI 0.33 to 1.45) Second Post-Test: **All 25 (8%) aOR = 0.48 (95% CI 0.27 to 0.86)** **Fast track 11 (7%) aOR = 0.43 (95% CI 0.21 to 0.91)** Standard 14 (9%); aOR = 0.52 (95% CI 0.26 to 1.04) Duration of total diagnostic procedure: Pretest: Hospital 1: 44.5(24.4) Hospital 2: 53.7(22.7) First Post-test: Hospital 1: **All 37.6 (SD 23.7) Fast track 17.4 (SD 9.7)***Standard 51.2 (SD 20.4)* Hospital 2: **All 31.7 (SD 26.3) Fast track 17.7 (SD 12.7)***Standard 59.2 (SD 24.6)* Second Post-test: Hospital 1: **All 41.8 (SD 21.9) Fast Track 32.5 (SD 14.4)** Standard: 49.5(SD 24.1) Hospital 2: **All 47.5 (SD 39.4) Fast Track 24.2 (SD 12.3)***Standard 79.7 (SD 41.3*) Bolded indicate significant decrease in mean days compared to pretest Italicized indicate significant increase in mean days compared with pretest	
Hahne, 2017 [27]	Proportion of Patients Receiving Co-Interventions (%) of General Medical Practitioner Visits 0–10 weeks: Intervention 12/28 (43%), Control 12/26 (46%), Risk difference −3%, 95% CI −28 to 22%, Relative risk 0.9, 95% CI 0.5 to 1.7, *P* = .81 11–52 weeks: Intervention 4/28 (14%), Control 12/26 (46%), Risk difference −32%, 95% CI −52 to −7%, Relative risk 0.3, 95% CI 0.1 to 0.8**,*P*= .01** Total: Intervention 12/28 (43%), Control 15/26 (58%), Risk difference −15%, 95% CI −38 to 11%), Relative risk 0.7, 95% CI 0.4 to 1.3, *P* = .28 Co-intervention Sessions Attended: Median (25th to 75th percentile) of General Medical Practitioner Visits 0–10 weeks: Intervention group 0.0 (0.0 to 2.0), Control group: 0.0 (0.0–2.0), *P* = .88 11–52 weeks: Intervention group: 0.0 (0.0 to 0.2), Control group 0.0 (0.0 to 2.8), ***P*< .01** Total: Intervention group 0.0 (0.0 to 2.0), Control group 1.0 (0.0 to 4.0), *P* = .17 Proportion of Patients Receiving Co-Interventions (%) of Any Other Healthcare Intervention Apart from Medical Practitioner 0–10 weeks: Intervention 7/28 (25%), Control 15/26 (458%), Risk difference −33% (95% CI −54 to −7%), Relative risk 0.4 (95% CI 0.2 to 0.9), ***P*= .02** 11–52 weeks: Intervention 9/28 (32%), Control 15/26 (58%), Risk difference −26% (95% CI −48 to 1%), Relative risk 0.6 (95% CI 0.3 to 1.1), *P* = .06 Total: Intervention 10/28 (36%), Control 21/26 (81%), Risk difference −41% (95% CI −61 to −15%), Relative risk 0.5 (95% CI 0.3 to 0.8), ***P*< .01** Co-intervention Sessions Attended: Median (25th to 75th percentile) of Any Other Healthcare Intervention Apart from Medical Practitioner 0–10 weeks: Intervention 0.0 (0.0 to 0.03), Control 1.0 (0.0–2.3), ***P*= .01** 11–52 weeks: Intervention 0.0 (0.0 to 5.0), Control 2.0 (0.0 to 10.8), *P* = .11 Total: Intervention 0.0 (0.0 to 2.8), Control 2.0 (1.0 to 16.6), ***P*<.01**	Visits: Intervention 9.4 (SD 1.6) Control 1.8 (SD 0.4)
**Neck pain**		
Cote, 2019 [28]	Number of Visits: Mean Weeks 1–3: Government guideline 3.8 (SD 2.3) Preferred-provider 2.7 (SD 1.9) Weeks 4–6: Government guideline 2.8 (SD 2.4) Preferred-provider 2.7 (SD 2.5) GP Education and Activation: mean 1.5 visits to GP in first 6 weeks (SD 0.8) GP Visits in the first 6 weeks: Government guideline 27.5% Preferred-provider 43.8% GP Education and activation 34.8%	Physiotherapy as Co-Intervention within the first 6 weeks: Government guideline 3.8% (1.3; 10.5) Preferred-provider 6.9% (4.7; 18.5) GP education and activation 14.5% (8.1; 24.7)
Horn, 2016 [19]	Adherent care: attended 46% fewer visits to health care providers. adjusted mean difference = 7.26 visits; IRR = 0.54, 95% CI 0.47 to 0.62; ***P*< .001**	Adherent care: attended 54% fewer visits for PT during an episode of care, adjusted mean difference = 3.63 visits; IRR = 0.44, 95% CI 0.36 to 0.55; ***P*<.001**

Bolded indicates statistical significance

*PT* physical therapy, *SE* standard error, *SD* standard deviation, *aOR* adjusted odds ratio

Two studies [31, 34] involved care pathways that encouraged early utilization of PT. Non-PT healthcare visits were reduced in both studies. One of the studies [34] found no differences in the number of PT visits; however, the duration of care was decreased for those that received guideline-based care. Combining guideline-based advice with an individualized functional restoration program resulted in significantly less non-PT healthcare visits in one study [27] but the number of PT visits to support this protocol was significantly higher. HCU rates did not appear to be significantly impacted by factors such as guideline delivery (active versus standard) [25], the addition of intensive group training [24] or symptom chronicity (acute versus chronic) [21]. There was also no significant difference between groups when comparing visits for a pain education group and guideline-based PT [26].

#### Imaging

Five studies [18, 19, 22, 24, 34] reported on utilization of imaging, as seen in Table 4. Three studies reported decreased imaging utilization rates for guideline adherent care compared to nonadherent care [18, 19, 22]. One study [34] found that participants in the Rapid Access program were less likely to have lumbar radiographs or advanced imaging over the 6-month follow-up period compared to nonparticipants. The addition of an intensive training protocol to guideline-based care did not significantly impact imaging utilization [24].

**Table 4:** Reported additional interventions and associated costs by study

Author, Year	Imaging	Medication	Procedural Interventions
**Low back pain**			
Childs, 2015 [22]	Adherent 17.0% Nonadherent 22.7% aOR = 0.72, (99% CI 0.69 to 0.76) Early 11.9% Delayed 21.0% aOR = 0.52, (99% CI 0.50 to 0.54)	Prescription Medication Costs: Adherent Care $886.27 (SE 19.82) Nonadherent Care $1233.90 (SE 19.86) Difference $347.63 (95% CI 292.63 to 402.63) Early $772.20 (SE 13.00) Delayed $762.74 (SE 12.44) Opioid Medication Use: Adherent 65.2% Nonadherent 66.0% Early 59.7% Delayed 0.3% aOR = 0.62, (99% CI 0.60 to 0.64)	Lumbar Spinal Injections: Adherent 11.7% Nonadherent 13.8% aOR = 0.82, (99% CI 0.77 to 0.87) Early 8.7% Delayed14.6% aOR = 0.56, (99% CI 0.53 to 0.59) Lumbar Spine Surgery: Adherent 2.6% Nonadherent 3.0% aOR = 0.85, (99% CI 0.75 to 0.96) Early .9% Delayed 3.2% aOR = 0.59, (99% CI 0.54 to 0.65)
Fritz, 2012 [16]		Opioid medication use All 49.1% Early 49.1% Delayed 55.3% OR = 0.78, (95% CI 0.66 to 0.93) Adherent 49.6% Nonadherent 53.2% Prescription Medication costs: All 104.23 (SE 3.01) Early $80.41 (SE 10.22) Delayed $116.83 (SE 11.27) Adherent $76.43 (SE 9.85) Nonadherent $98.85 (SE 9.61)	Lumbar spine surgery All 2.5% Early 4.7% Delayed 9.9% OR = 0.45, (95% CI 0.32 to 0.64) Adherent 5.1% Nonadherent 8.1% OR = 0.61; (95% CI 0.38 to 0.98) Lumbar spinal injections All 7.1% Early 10.1% Delayed 21.2% OR = 0.42 (95% CI 0.32 to 0.64) Adherent 12.6% Nonadherent 17.8% OR = 0.55, (95% CI 0.48 to 0.91) Costs: Surgical/Injection Procedures: All $740.44 (SE 36.84) Early $1018.88 (170.65) Delayed $2760.62 (SE 381.27) Adherent $1445.23 (SE 486.37) Nonadherent $1965.72 (SE 229.42)
Hoeijenbos, 2005 [25]		Prescribed medicines: Baseline 40% 12 weeks 10% Not prescribed medicines: Baseline 49% 12 weeks +/− 23% Mean direct medical costs for the previous 6 weeks Baseline: Intervention € 7 median € 1, Control € 7, median €10 6 weeks: Intervention € 4, median € 0, Control € 3, median € 0 12 weeks: Intervention € 1, median € 0, Control € 3, median € 0 26 weeks: Intervention € 3, median € 0, Control € 3, median € 0 52 weeks: Intervention € 3, median € 0, Control € 3, median € 0 Percentage utilizing and mean cost of prescribed medicines by doctor in previous 6 weeks: Baseline: Intervention 41.5%, € 5.73, median € 0; Control 37.8%, € 5.29, median € 0 6 weeks: intervention **21.4%, € 3.42,** median € 0; Control **20.8%, € 2.31**, median € 0 12 weeks: Intervention **10.4%, € 1.19,** median € 0, Control **10.8%**, € 1.90, median € 0 26 weeks: Intervention 8.8%, € 2.67, median € 0, Control 8.7%, € 1.87, median € 0 52 weeks: Intervention 11.7%, € 2.20, median € 0, Control 9.9%, € 2.19, median € 0 Percentage utilizing and mean cost nonprescription medications in previous 6 weeks: Baseline: Intervention 49.0%, € 1.63, median € 0; Control 49.0%, € 1.88, median € 0 6 weeks: intervention **30.8%, € 0.77,** median € 0; Control **38.5%**, € 1.16, median € 0 12 weeks: Intervention **23.5%**, € 0.46, median € 0, Control **22.1%**, € 0.79, median € 0 26 weeks: Intervention 20.5%, € 0.24, median € 0, Control 18.3%, € 0.61, median € 0 52 weeks: Intervention 23.4%, € 0.78, median € 0, Control 20.3%, € 0.59, median € 0 Bolded indicates significant difference from previous measure	
Magel, 2018 [34]	Radiographs: All patients 214 (53.5%) Participants 32 (25.8%) Nonparticipants 182 (65.9%) Refused 71 (70.3%) Not offered 111 (63.4%) Advanced Imaging: All patients 85 (21.3%) Participants 11 (8.9%) Nonparticipants 79 (27.2%) Refused 26 (25.7%) Not offered 53 (28.5%) Participants were less likely to have lumbar radiographs and advanced imaging over the 6-month follow period compared to nonparticipants ***P*< .05**		Epidural steroid injections: All patients 90 (22.5%), Participants 10 (8.1%), Nonparticipants 80 (29.0%), Refused 30 (29.7%), Not offered 50 (28.6%) Spinal surgical procedure: All patients 9 (2.3%), Participants 2 (1.6%), Nonparticipants 7 (2.5%), Refused 2 (2.0%), Not offered 5 (2.8%) Participants were less likely to have epidural injections over the 6 month follow period compared to nonparticipants ***P*< .05**
Sharma, 2019 [26]		Number of NSAIDs per week used at follow-up: Pain Education 2 Control 5	
**Acute low back pain**			
Fritz, 2008 [18]	Use of diagnostic procedures: Adherent 14.4%, Nonadherent 23.6%; aOR = 0.53, (95% CI 0.31 to 0.92); ***P*<.05**. Use of MRI: Adherent 8.3%, Nonadherent 15.9%; aOR = 0.47, (95% CI 0.24 to 0.94); ***P*<.05**	Use of prescription medication: Adherent 46.2%, Nonadherent 57.2%, aOR = 0.64 (95% CI 0.43 to 0.97); ***P*< .05** Use of skeletal muscle relaxants: Adherent 16.7%, Nonadherent 25.4%, aOR = 0.59, (95% CI 0.35 to 0.99); ***P*< .05**	Injection procedures: Adherent 9.1%, Nonadherent 15.9%; aOR = 0.40, (95% CI 0.18 to 0.85); ***P*< .05** Epidural injection with fluoroscopy: Adherent 5.3%, Nonadherent 12.1%; aOR = 0.40, (95% CI 0.18 to 0.94); ***P*< .05**
**Chronic low back pain**			
Van der Roer, 2008 [24]	X-ray: Protocol 0.2 (SD 0.8), 9.1% Guideline 0.3 (SD 1.0), 10.6% MRI: Protocol 0.2 (SD 0.6), 7.3% Guideline 0 (SD 0.3), 2.1% CT-scan: Protocol 0 (SD 0), 0% Guideline 0 (SD 0), 2.1%	Medication (% yes): Protocol 56.7% Guideline 55.6% Medication Costs: Protocol € 12 (SD 23) Guideline € 13 (SD 24) Mean Difference € −1, (95% CI −11 to 8)	
**Low back pain with radicular symptoms**			
Hahne, 2017 [27]		No significant differences aside from proportion taking Paracetamol at 12 months Intervention 2/28 (7%) Control 7/25 (28%) Risk difference −21%, 95% CI −41 to −0.2%	No participants underwent surgery Epidural injection: Intervention 2 Control 1 Medial Branch Block: Intervention 0 Control 1
**Neck pain**			
Horn, 2016 [19]	Adherent care: had 43% fewer diagnostic images. adjusted mean difference = 0.43 images, IRR = 0.57, 95% CI 0.36 to 0.90; ***P*= 0.02**	Adherent care: 25% fewer prescription medications. adjusted mean difference = 1.00 prescription; IRR = 0.75, 95% CI 0.59 to 0.95; ***P*= .02**	

Bolded indicates statistical significance

*PT* physical therapy, *SE* standard error, *SD* standard deviation, *aOR* adjusted odds ratio

#### Medication

Full results on medication utilization and costs can be found in Table 4. Eight studies [16, 18, 22, 24–27] reported on medication utilization and/or cost. Guideline adherence resulted in decreased prescription medication costs in one study [22], significantly fewer prescription medications in two studies [18, 19], and decreased skeletal muscle relaxants in one study [18]. The addition of an intensive group training protocol to guideline-based care [24] and method of guideline delivery (active versus standard) [25] did not significantly impact medication use. Similarly, there was no significant difference in medication use between groups receiving individualized functional restoration and guideline-based advice aside from the proportion taking Paracetamol at 12 months [27]. Statistical analysis was not performed in two studies thus limiting comparisons [16, 26].

#### Procedures

Five studies [16, 18, 22, 27, 34] reported on procedure utilization, as demonstrated in Table 4. Patients receiving guideline adherent care were significantly less likely to have diagnostic procedures, injection procedures, and epidural injections with fluoroscopy in one study [18], and had decreased likelihood of surgery and receiving injection in another study [16]. A third study [22] reported that guideline adherent care resulted in decreased lumbar spine injections and lumbar spine surgeries. Magel et al. [34] found participants receiving PT via Rapid Access were significantly less likely to receive epidural steroid injections compared to nonparticipants. Statistical analysis was not performed in one study [27].

### Pain

Twelve studies [17–19, 23, 24, 26–30, 32, 35] included assessment of pain (Table 5). The primary outcome measure was the numeric pain rating scale (NPRS) or numeric rating scale (NRS), utilized in all but one study [35]. Five studies [17–19, 23, 35] compared pain outcomes with guideline adherence. Two of the studies [17, 18] found a statistically significant difference favoring adherent care. Schroder et al. [23] found similar results at 3 and 6 months but no difference between groups at 12 months. Rutten et al. [35] found no association between percentage of guideline adherent care and pain with the exception of the chronic low back pain subgroup, which demonstrated a medium to large negative correlation. One study [19] found a statistically significant difference favoring nonadherent care.

**Table 5:** Reported pain outcomes by study

Author, Year	Measure	Results
**Low back pain**		
Bekkering, 2005 [29]	NRS	Pain improved in both groups over initial 12 weeks Baseline: Intervention 7.0, IQR 5.0–8.0; Control 7.0, IQR 5.0–8.0 6 week: Intervention 3.0, IQR 2.0–5.0; Control 3.0, IQR 2.0–5.0 12 week: Intervention 2.0, IQR 1.0–4.0; Control 2.0, IQR 1.0–4.0 26 week: Intervention 2.0, IQR 1.0–4.0; Control 1.0, IQR 0.0–4.0 52 week: Intervention 2.0, IQR 0.0–4.0; Control 1.0, IQR 0.3–3.0 At 12 weeks: difference in pain intensity was 0.34, (95% CI −0.19 to 0.88) No difference between groups over the 12 months. (*X*^2^=6.05, df=4, *P*>.05)
Kongsted, 2019 [32]	NRS	Change Scores baseline to 4 months: Before 0.6 (95%CI −0.05 to 1.3) After 1.9 (95%CI 1.2 to 2.7) GLA:D 1.2 (95%CI 0.6 to 1.7)
Lemieux	NRS	Back Pain: Pre-training median 5, (Q1, Q3 3,7) Post-training median 3 (Q1, Q3 1,4) Difference in median −2, ***P*<.001** Leg Pain: Pre-training median 2 (Q1,Q3 0.5,5.0) Post-training median 1 (Q1, Q3 0,3) Difference in median −1, ***P*<.001**
Rutten, 2010 [35]	VAS	Association between % Guideline adherence and VAS Average: B = −0.17, Beta= −0.07, *P*=.499 Correlation of Adherence with VAS Average for Subgroups: Acute −.06, *P*>.05 Subacute −.14, *P*>.05 Chronic −.45, ***P*<.01**
Sharma, 2019 [26]	PROMIS Pain Intensity (NRS)	PROMIS short form pain intensity: PEG Change 5.28, (95% CI 2.91 to 7.65), ***P*<.001** CG Change 1.72, (95% CI −0.82 to 4.26) *P*>.05 Between groups: *t* = 2.16, difference 3.56, (95% CI 0.21 to 6.91)**,*P*<.05** PROMIS short form pain interference: PEG Change 4.47, (95% CI 1.191 to 7.04), ***P*<.001** CG Change 3.03, (95% CI 0.69 to 5.36), ***P*<.05** Between groups: *t* = 0.88, difference 1.45, (95% CI −1.90 to 4.79), *P>*.05
Schroder, 2021 [23]	NRS	Between-Group Effects Adherent/Nonadherent Care Baseline: Non CPQI 6.3 (5.5 to 7.1) CPQI Adherent 6.1 (5.4 to 6.9) 3 months: Non CPQI Adherent −2.5 (95% CI −3.0 to −2.0) ***P*< .001**; CPQI Adherent −3.4 (95% CI −4.0 to −2.8) ***P*< .001;** Between-Group Effect 0.9 (95% CI 0.3 to 1.6) ***P*= .004** 6 months: Non CPQI Adherent −2.1 (95% CI −2.7 to −1.5) ***P*< .001;** CPQI Adherent −3.2 (95% CI −3.8 to −2.6) ***P*< .001;** Between-Group Effect 1.1 (95% CI 0.4 to 1.8) ***P*= .002** 12 months: Non CPQI Adherent −2.6 (95% CI −3.2 to −2.0) ***P*< .001;** CPQI Adherent −3.1 (95% CI −3.7 to −2.5) ***P*< .001;** Between-Group Effect 0.5 (95% CI −0.2 to 1.2) *P* = .169 Between-Group Effects of Control and Intervention Group Baseline: Control 6.1 (5.6 to 6.7), Intervention 6.4 (5.7 to 7.0) 3 months: Control −2.6 (95% CI −3.1 to −2.1) ***P*< .001;** Intervention −2.9 (95% CI −3.4 to −2.5) ***P*< .001** Between-Group Effect −0.3 (95% CI −0.3 to 0.9) *P* = .263 6 months: Control −2.4 (95% CI −3.0 to −1.8) ***P*< .001;** Intervention −2.7 (95% CI −3.2 to −2.2) ***P*< .001;** Between-Group Effect −0.3 (95% CI −0.3 to 0.9) *P* = .357 12 months: Control −3.1 (95% CI −3.7 to −2.5) ***P*< .001** Intervention −2.8 (95% CI −3.3 to −2.3) ***P*< .001;** Between-Group Effect −0.3 (95% CI −0.9 to 0.3) *P* = .297 Bonferroni corrected *P* value of *P* < .017
**Acute low back pain**		
Fritz, 2007 [17]	NPRS	Adherent vs nonadherent care 22.4% mean difference in improvement, (95% CI 17.5 to 27.3), ***P*<.001** Change in pain rating: All 3.0 (SD 2.7) Adherent 3.6 (SD 2.8) Nonadherent 2.6 (SD 2.7) Percentage change in pain rating, Between groups: ***P<*.05** All 47.1% (SD 43.5) Adherent 60.5% (SD 39.1) Nonadherent 38.0% (SD 44.1)
Fritz, 2008 [18]	NPRS	Percent change in pain rating: mean difference 11.3% (95% CI 1.6 to 20.9), ***P*<.05** Adherent 49.1% (SD 45.9) Nonadherent 39.2% (SD 46.8)
**Chronic low back pain**		
Van der Roer, 2008 [24]	NRS	No significant difference between groups: −1.02 points; (95% CI −2.14 to 0.09)
**Low back pain with radicular symptoms**		
Hahne, 2017 [27]	NRS	All groups improved Back pain: (SMD=standardized mean difference) 5-week NRS: Intervention 3.1(SD 2.2), Control 3.5 (SD 2.5), Adjusted SMD 0.1 (95% −0.4 to 0.6) Adjusted between-group difference 0.2 (95% CI −1.0 to 1.5) *P*=.72 10-week NRS: Intervention 2.4 (SD 1.6), Control 4.0 (SD 2.6), Adjusted SMD 0.7 (95% CI 0.1 to 1.2), Adjusted between-group difference 1.4 (95% CI 0.2 to 2.7) ***P*=.02** 26-week NRS: Intervention 2.4 (SD 1.6), Control 3.5 (SD 2.6), Adjusted SMD 0.4 (95% CI −0.1 to 0.9), Adjusted between-group difference 0.9 (95% CI −0.3 to 2.2) *P*=.13 52-week NRS: Intervention 2.4 (SD 2.0), Control 3.6 (SD 2.5), Adjusted SMD 0.5 (95% CI −0.1 to 1.0), Adjusted between-group difference 1.1 (95% CI 0.2 to 2.3) *P*=.9 Leg pain**:** 5-week NRS: Intervention 3.6 (SD 2.4), Control 4.4 (SD 3.0), Adjusted SMD 0.4 (95% CI −0.2 to 0.9), Adjusted between-group differences 1.0 (95% CI −0.4 to 2.3) *P*=.16 10-week NRS: Intervention 2.9 (SD 2.3), Control 3.8 (SD 3.0), Adjusted SMD 0.4 (95% CI −0.2 to 0.9), Adjusted between-group difference 1.1 (95% CI −0.3 to 2.4) *P*=.13 26-week NRS: Intervention 2.0 (SD 2.1), control 3.0 (SD 2.9), Adjusted SMD 0.5 (95% CI −0.1 to 1.0), Adjusted between-group difference 1.2 (95% CI −0.2 to 2.6) *P*=.09 52-week NRS: Intervention 2.1 (SD 2.4), Control 2.9 (SD 2.8), Adjusted SMD 0.3 (95% CI −0.2 to 0.9), Adjusted between-group difference 0.9 (95% CI −0.5 to 2.3) *P*=.21
**Neck Pain**		
Cote, 2019 [28]	NRS	NRS improved within all groups but no differences between groups (*P*>.05) Baseline: Government guideline 5.6 (SD 2.1), Preferred-provider 5.7 (SD 2.0), Education and activation 5.9 (SD 2.1) 6 weeks: Government guideline 2.7 (95% CI 2.1 to 3.3), Preferred-provider 2.2 (95% CI 1.6 to 2.8), GP Education and activation 2.4 (95% CI 1.7 to 3.0) 3 months: Government guideline 3.5 (95% CI 2.9 to 4.0), Preferred-provider 3.3 (95% CI 2.7 to 3.9), GP Education and activation 3.3 (95% CI 2.6 to 3.9) 6 months: Government guideline 3.4 (95% CI 2.8 to 4.1), Preferred-provider 3.2 (95% CI 2.5 to 3.8), GP Education and activation 3.6 (95% CI 3.0 to 4.3) 9 months: Government guideline 3.7 (95% CI 3.1 to 4.3), Preferred-provider 4.0 (95% CI 3.4 to 4.5), GP Education and activation 3.8 (95% CI 3.1 to 4.4) 12 months: Government guideline 3.6 (95% CI 3.0 to 4.2), Preferred-provider 3.2 (95% CI 2.6 to 3.8), GP Education and activation 3.6 (95% CI 2.9 to 4.2)
Horn, 2016 [19]	NPRS	The nonadherent group demonstrated greater percentage improvement in pain. ***P*=.01** Adherent 7.04 (95% CI −11.73 to 25.70) Nonadherent 33.11 (95% CI 25.99 to 40.22)

Bolded indicates statistical significance

*DF* degrees of freedom, *NRS/NRP* Numeric Rating Scale/Numeric Pain Rating Scale, *VAS* visual analog scale, *SD* standard deviation

Sharma et al. [26] reported that the addition of pain education to guideline-based care resulted in significantly less pain compared to guideline-based care alone. Hahne et al. [27] found that the addition of individualized functional restoration training to guideline-based advice resulted in significantly lower back pain at 10 weeks but no difference between groups at the end of year 1. Three studies [24, 28, 29] found no statistically significant differences between groups for pain outcomes when comparing method of guideline delivery [29], government-regulated guidelines [28], and addition of intensive training to guideline-based care [23]. Two studies [30, 32] did not assess statistical significance across groups.

### Physical functioning and disability

Fourteen studies [17–19, 22–30, 32, 35, 36] reported patient-level outcomes related to function; for full results refer to Table 6. The most prevalent functional measure utilized was the Modified Oswestry Low Back Pain Disability Index (ODI). Guideline adherent care resulted in significant improvement in function in four of five studies [17, 18, 30, 35] that assessed significance in patients with LBP when utilizing the ODI as the primary measure. However, one [30] did not utilize a control or comparator. Guideline adherence did not significantly impact function in one study that included patients with neck pain [19].

**Table 6:** Reported physical functioning and disability outcomes by study

Author, Year	Outcome Measure	Results
**Low back pain**		
Bekkering, 2005 [29]	QBPDS	Physical Functioning improved in both groups (Passive implementation vs Active+Passive implementation). No difference between groups at any time point over 12 months (*Χ*^2^ 4.88, df=4, *P* > 0.05). Baseline: Intervention 38.0 (IQR 26.5 to 50.5), Control 40.5 (IQR 26.3 to 55.8) 6 weeks: Intervention 24.0 (IQR 13.0 to 40.0), Control 23.5 (IQR 11.0 to 37.8) 12 weeks: Intervention 20.0 (IQR 7.0 to 32.8), Control 17.5 (IQR 6.0 to 30.8) 26 weeks: Intervention 16.0 (IQR 5.0 to 32.0), Control 11.0 (IQR 4.0 to 29.0) 52 weeks: Intervention 17.0 (IQR 4.6 to 32.0), Control 13.0 9 (IQR 4.8 to 29.0)
Hoeijenbos, 2005 [25]	EQ-5D	Baseline: intervention 0.6730 (SD 0.2042), Control 0.6134 (0.2661) *P* = .006 Lower self-care score at baseline in control group (values not provided) 6 weeks: intervention 0.7778 (SD 0.1978), Control 0.7497 12 weeks: intervention 0.8141 (SD 0.1988), Control 0.7873 (SD 0.2210) No significant difference from 6 weeks onwards between groups
Karlen, 2015 [36]	ODI	Change in ODI per visits 25.2 to 31.5% Ave improvement in ODI/visit improved form 3.8% to 5.8% Mean % ODI improvement: 2010: 25.2% 2011: 28.5% 2012: 30.4% 2013: 32.9% 2014: 31.5% Mean % ODI Improvement per visit: 2010: 3.8% 2011: 4.5% 2012: 5.1% 2013: 5.4% 2014: 5.8%
Kongsted, 2019 [32]	ODI	ODI: Unadjusted: Before 1.8 (95% CI −1.2 to 4.8); After 4.4 (95% CI 1.7 to 7.1); GLA:D 6.5 (95% CI 4.6 to 8.4) Adjusted: Before 2.4 (95% CI −0.5 to 5.3); After 4.8 (95% CI 1.9 to 7.6); GLA:D 5.7 (95% CI 3.3 to 8.1)
Lemieux, 2021 [30]	Perceived Fitness, ODI	Perceived Physical Fitness Pre-training median: 19, (Q1, Q3 16, 24); Post-training median 22 (Q1, Q3 15, 27); Difference in median 3, *P*=.031 ODI Pre-training median 25, (Q1, Q3 16, 34); Post-training median 20 (Q1, Q3: 10, 28); Difference in median −5, ***P*<.001**
Rutten, 2010 [35]	QBPDS	Association between % Guideline Adherence and QBPDS B= −0.35, Beta= −0.21, *P* = −.023 Significant Associations between Percentage of Adherence to Individual Steps of the Process and QBPDS: History taking −0.16, *P*< .1 Analysis −0.17, ***P*<.05** Evaluation −0.30, ***P*<.001** Correlation of Adherence with QBPDS in subgroups: Acute −.20, *P*>.05 Subacute −.15, *P*>.05 Chronic −.38, ***P*<.05**
Sharma, 2019 [26]	PROMIS; 2-item Quality of Life	PROMIS short form sleep disturbance: PEG Change (95% CI) 7.62 (95% CI 3.50 to 11.74), ***P*< .01** CG Change 3.49 (95% CI −0.12 to 7.10) *P* > .05 Between groups: *t* = 1.58, difference 4.13 (95% CI −1.16 to 9.42) *P* > .05 2-item Quality of Life change: PEG change −0.79 (95% CI −1.42 to −0.15), ***P*< .05** CG change −0.47 (95% CI −1.04 to 0.09) *P* > .05 Between groups: *t* = −0.78, difference −0.32 ( 95% CI −1.13 to 0.50) *P >* .05
Schroder	ODI, EQ-5D	Between-group difference for patients receiving CPQI adherent/Nonadherent care: ODI: Baseline: Non CPQI Adherent 32.4 (95% CI 27.5 to 37.3); CPQI Adherent 28.3 (95% CI 23.5 to 33.2) 3 months: Non CPQI Adherent −9.0 (95% CI −11.8 to −6.2) ***P*< .001;** CPQI Adherent−11.3 (95% CI −14.2 to −8.3) ***P*< .001;** Between-Group Effect 2.3 (−1.1 to 5.6) *P* = .178 6 months: Non CPQI Adherent −8.9 (95% CI −12.1 to −6.0) ***P*< .001;** CPQI Adherent −12.7 (95% CI −16.1 to −9.4) ***P*< .001;** Between-Group Effect 3.8 (0.3 to 7.6) *P* = .048 12 months: Non CPQI Adherent −10.7 (95% CI −13.9 to −7.6) ***P*< .001;** CPQI Adherent−13.2 (95% CI −16.5 to −9.8) ***P*< .001;** Between-Group Effect 2.4 (−1.4 to 6.2) *P* = .207 EQ-5D: Baseline: Non CPQI Adherent 0.51 (95% CI 0.45 to 0.57); CPQI Adherent 0.59 (95% CI 0.52 to 0.65) 3 months: Non CPQI Adherent 0.12 (95% CI 0.05 to 0.18) ***P*< .001;** CPQI Adherent 0.15 (95% CI 0.09 to 0.22) ***P*< .001;** Between-Group Effect −0.03 (95% CI −0.11 to 0.03) *P* = .294 6 months: Non CPQI Adherent 0.14 (95% CI 0.08 to 0.20) ***P*< .001;** CPQI Adherent 0.19 (95% CI 0.13 to 0.26) ***P*< .001;** Between-Group Effect −0.05 (95% CI −0.12 to 0.02) *P* = .161 12 months: Non CPQI Adherent 0.19 (95% CI 0.13 to 0.25) ***P*< .001;** CPQI Adherent 0.19 (95% CI 0.12 to 0.25) ***P*< .001;** Between-Group Effect 0.00 (95% CI −0.07 to 0.07) *P* = .985 Between-Group Effects for outcomes in Control and intervention group: ODI: Baseline: Control 31.6 (95% CI 27.2 to 36.1) Intervention 30.4 (95% CI 25.6 to 35.3) 3 months: Control −10.5 (95% CI −13.4 to −7.6) ***P*< .001**; Intervention −8.7 (95% CI −11.2 to −6.2) ***P*< .001;** Between-Group Effect −1.8 (−5.0 to 1.3) *P* = .248 6 months: Control −10.9 (95% CI −14.1 to −7.7) ***P*< .001**; Intervention −10.2 (95% CI −12.9 to −7.5) ***P*< .001**; Between-Group Effect −0.7 (95% CI −4.2 to 2.7) *P* = 0.674 12 months: Control −14.2 (95% CI −17.3 to −11.1) ***P*< .001**; Intervention −11.3 (95% CI −13.9 to −8.6) ***P*< .001**; Between-Group Effect −3.0 (−6.3 to 0.4) *P* = .081 EQ-5D index: Baseline: Control 0.55 (95% CI 0.50 to 0.60); Intervention 0.52 (95% CI 0.46 to 0.58) 3 months 0.12 (95% CI 0.06 to 0.18) ***P*< .001**; Intervention 0.15 (95% CI 0.10 to 0.21) ***P*< .001**; Between-Group Effect −0.03 (95% CI −0.10 to 0.04) *P* = .381 6 months: Control 0.13 (95% CI 0.07 to 0.19) ***P*< .001**; Intervention 0.20 (95% CI 0.15 to 0.25) ***P*< .001**; Between-Group Effect −0.07 (95% CI −0.14 to −0.01) *P* = .034 12 months: Control 0.19 (95% CI 0.13 to 0.25) ***P*< .001**; Intervention 0.20 (95% CI 0.14 to 0.25) ***P*< .001**; Between-Group Effect −0.01 (95% CI −0.07 to 0.06) *P* = .838 * Bonferroni corrected significance thresholds of *P* ≤ 0.017
**Acute low back pain**		
Fritz, 2007 [17]	Modified ODI	All patients 47.9% (570) achieved at least 50% improvement. Between groups, achieved at least 50% improvement: Adherent 64.7%; Nonadherent 36.5% ***P*< 0.001** Change in Oswestry: All 19.8 (SD 18.3); Adherent 25.1 (SD 18.3); Nonadherent 16.3 (SD 17.5) Percent Change in Oswestry: All 44.9% (SD 37.7); Adherent 59.4% (SD 35.2); Nonadherent 35.1% (SD 36.1)
Fritz, 2008 [18]	Modified ODI	Percent change in ODI: Adherent group 53.7% (SD 33.1), Nonadherent group 37.5% (SD 33.3), ***P*< .05** **Mean difference 16.2%, (95% CI 9.5 to 22.9)** Successful outcome of physical therapy (achieving at least 50% improvement on the OSW-disability score): Adherent 59.1%, Nonadherent 37.8%, ***P*< .05.**
**Chronic low back pain**		
Van der Roer, 2008 [24]	RMDQ, EQ-5D	RMDQ: No statistically significant differences between groups Function 0.06 points (95% CI −2.22 to 2.34) EQ-5D No statistically significant difference between groups QALYs 0.03 (95% CI −0.06 to 0.12).
**Low back pain with radicular symptoms**		
Hahne, 2017 [27]	Modified ODI	Baseline: Intervention 36.8 (SD 14.1), Control 37.5 (SD 16.1) 5 weeks: Intervention 27.4 (SD 15.5), Control 28.5 (SD 17.7), Adjusted SMD 0.0 (95% CI −0.5 to 0.6), Adjusted between-group difference 0.4 (95% CI −7.0 to 7.8) *P* = .92 10 weeks: Intervention 20.5 (SD 12.9), Control 28.9 (SD 21.6), Adjusted SMD 0.4 (95% CI −0.1 to 1.0), Adjusted between-group difference 7.7 (95% CI 0.3–15.1) ***P*= .04** 26 weeks: Intervention16.4 (SD 13.0), Control 22.8 (SD 19.9), Adjusted SMD 0.3 (95% CI −0.2 to 0.9), Adjusted between-group difference 5.7 (95% CI −1.7 to 13.1) *P* = .13 52 weeks: Intervention 14.2 (SD 15.4), Control 22.9 (SD 21.2), Adjusted SMD 0.4 (95% CI −0.1 to 1.0), Adjusted between-group difference 8.2 (95% CI 0.7–15.6) ***P*= .03**
**Neck pain**		
Cote, 2019 [28]	Whiplash Disability Questionnaire, SF-36	Whiplash Disability Questionnaire: 6 weeks: Government guideline 0.0 (95% CI −8.4 to 8.4), Preferred-provider 0.2 (95% CI −9.2 to 9.5), GP Education and activation 0.2 (95% CI − 8.7 to 9.0) 3 months: Government guideline 3.0 (95% CI −6.2 to 12.2), Preferred-provider −1.1 (95% CI −10.9 to 8.7), GP Education and activation 1.9 (95% CI −7.5 to 11.2) 6 months: Government guideline −5.5 (95% CI −15.9 to 4.9), Preferred-provider −2.7 (95% CI −13.2 to 7.8), GP Education and activation −8.2 (95% CI −18.7 to 2.2) 9 months: Government guideline −1.8 (95% CI −13.2 to 9.6), Preferred-provider 2.8 (95% CI −8.7 to 14.3), GP Education and activation 1.0 (95% CI −10.1 to 12.0) 12 months: Government guideline −4.8 (95% CI −15.2 to 5.6), Preferred-provider 3.3 (95% CI −7.3 to 14.0), GP Education and activation −1.5 (95% CI −12.3 to 9.3) No difference between groups (*P* > 0.05) SF-36 Physical Component: 6 weeks: Government guideline 0.4 (95% CI −2.8 to 3.7), Preferred-provider 0.2 (95% CI −3.2 to 3.5), GP Education and activation 0.6 (95% CI −2.4 to 3.7) 3 months: Government guideline 0.4 (95% CI −3.0 to 3.9), Preferred-provider −0.2 (95% CI −3.9 to 3.5), GP Education and activation 0.2 (95% CI −3.1 to 3.5) 6 months: Government guideline 0.4 (95% CI −3.0 to 3.8), Preferred-provider −1.0 (95% CI −5.0 to 2.9), GP Education and activation −0.7 (95% CI −4.4 to 3.1) 9 months: Government guideline 3.8 (95% CI −0.5 to 8.2), Preferred-provider −2.9 (95% CI −7.4 to 1.6), GP Education and activation 0.9 (95% CI −3.1 to 4.9) 12 months: Government guideline 1.6 (95% CI −2.0 to 5.1), Preferred-provider −2.1 (95% CI −6.1 to 2.0), GP Education and activation −0.5 (95% CI −4.4 to 3.4) No difference between groups (*P* > 0.05) SF-36 Mental Component: 6 weeks: Government guideline −3.3 (95% CI −7.4 to 0.9), Preferred-provider −0.8 (95% CI −4.9 to 3.2), GP Education and activation −4.1 (95% CI −8.4 to 0.3) 3 months: Government guideline −0.7 (95% CI −5.4 to 4.0), Preferred-provider −0.7 (−95% CI 5.3 to 4.0), GP Education and activation −1.3 (95% CI −6.2 to 3.6) 6 months: Government guideline 2.2 (95% CI −2.7 to 7.1), Preferred-provider 0.3 (95% CI −4.1 to 4.7), GP Education and activation 2.6 (95% CI −2.5 to 7.6) 9 months: Government guideline −0.3 (95% CI −6.1 to 5.5), Preferred-provider 1.8 (95% CI −3.6 to 7.2), GP Education and activation 1.5 (95% CI −4.1 to 7.1) 12 months: Government guideline −1.5 (95% CI −6.7 to 3.8), Preferred-provider −0.6 (95% CI −5.2 to 4.0), GP Education and activation −2.1 (95% CI −7.2 to 3.0) No difference between groups (*P* > 0.05)
Horn, 2016 [19]	NDI	No significant different between groups for disability score *P* = .32

Bolded indicates statistical significance

*SD* standard deviation, *QBPDS* Quebec Back Pain Disability Score, *ODI* Oswestry Disability Index, *EQ-5D* EuroQol-5D

One study [27] reported that the addition of an individualized restoration program to guideline-based advice resulted in significantly improved function at 10 weeks and 52 weeks compared to guideline-based advice alone. Sharma et al. [26] reported that the addition of pain education to guideline-based care resulted in significant improvement in sleep. There was no significant difference between groups when compared to guideline-based care alone. The remaining studies did not find significant between-group differences or did not include a control group, except for one [25] who reported baseline differences between groups which disappeared after 6 weeks.

## Discussion

The purpose of this systematic review was to determine the influence of guideline implementation on clinical outcomes of pain, physical function/disability, and HCU metrics in patients seeking physical therapy for neck and/or low back pain. Implementation strategies for CPGs were also examined to determine the variance in the focus of implementation strategies on success of the implementation. Our review identified a number of approaches to guideline implementation, with the most common implementation process being *Managing Quality*. This is largely due to the frequency with which “audit and provide feedback” was utilized, a discrete implementation strategy under the *Managing Quality* key implementation process. *Educating* was frequently utilized as many implementation methods included dissemination of guidelines and educational material. When we examined the relationship of implementation of CPGs and clinical outcomes, we found that, across studies, implementation and adherence to guidelines was beneficial for decreased HCU, including decreasing costs, total number of healthcare visits, medications, and procedural interventions. However, there were inconsistent findings for the benefit of guideline implementation for improvements in pain and function in patients. Pain improved in all groups studied, but results did not appear to favor guideline implementation. Full comparisons of physical function and disability outcomes were difficult to compare due to lack of consistency in measures utilized.

### Guideline implementation

Few studies assessed the effects of types of guideline implementation, or utilized controls or comparators for implementation strategy, and more evidence is needed to evaluate the most effective guideline implementation strategies to improve patient outcomes. Only two articles [25, 29] in our review, reporting on one study, assessed the impact of active versus passive guideline implementation and found no difference in patient outcomes between groups. When assessing HCU, the authors concluded that there may be some benefit to active implementation, but the results were small and did not lend clear support to use of an active strategy when considering the cost of implementation. Additional studies in this review could be classified as active, including engagement of stakeholders, or passive, including only dissemination of materials, implementation, but did not compare active and passive interventions within the same study.

Previous systematic reviews have identified active, multifactorial implementation strategies as more effective [37, 38]; however, a more recent review [39] found no benefit to an active over passive implementation. Additionally, a recent review by Mesner [40] indicated that discrete, utilizing only a singular implementation strategy, or multifaceted, utilizing multiple strategies implementation strategies may not be the best indicators of guideline uptake, rather the duration of the implementation program may better predict this. Powell et al. [14] propose an additional distinction when using more than one implementation strategy. The authors suggest distinguishing between multifaceted strategies and blended strategies, defined as multifaceted strategies that address “multiple levels or barriers to change” and are “packaged as a protocolized and branded implementation intervention” (p. 125). Implementation frameworks could be utilized to satisfy this definition.

The use of an implementation framework, model, or theory can aid in both intervention design and assessment of outcomes in the formulation of a blended implementation intervention [41]. Several included studies utilized published implementation frameworks to guide their intervention, while others utilized theories such as the behavior change theory, learning theory, and change management theory to structure their intervention. Utilizing a theory-informed implementation strategy is proposed to improve implementation outcomes [42] but often explanations for the theoretical basis are lacking [43]. In our review, few stated a rationale for their choice. To aid in this decision-making, implementation frameworks are often structured around different aims within the implementation process [41]. Some authors [44] suggest utilizing multiple frameworks for a more comprehensive approach, for example, using one framework for guiding the implementation process and one for evaluating outcomes.

The use of an implementation framework or model promoted the use of the strategy “tailor strategies to overcome barriers and honor preferences,” and studies utilizing this typically demonstrated improvement in outcomes over time in our review. This suggests this may be a beneficial strategy for implementation. The remaining included studies failed to incorporate assessment of barriers and subsequent tailored strategies. Comprehensive, blended strategies, including barrier assessment, may be beneficial as these may more efficiently address the obstacles and needs of the involved stakeholders and should be further investigated.

Audit and provide feedback was utilized in almost every included study. However, the majority of the studies utilized this method to assess adherence, rather than to inform clinicians of their progress. One study [45] suggests this strategy is most effective when employed in a timely, personalized, and non-punitive manner. Since the majority of studies utilizing this strategy did not provide individualized feedback, rather using the information as an aggregate measure, improvement in providers’ adherence and thus patient outcomes is less likely. Though we did not assess therapist adherence as a measure in our review, it is worth noting the impact this may have had on outcomes.

### Adherence

Implementation of guidelines does not guarantee provider adherence to guidelines or a change in provider practice. One study [46] utilizing an implementation framework by Grol et al. [47] found guideline adherence to increase frequent use of guidelines to only 55%, and a review by Al Zoubi et al. [37] indicated that multifaceted interventions may increase provider adherence, but with mixed effects. Factors that may influence uptake of CPGs include therapist beliefs and patient beliefs or expectations [48], and implementation strategies may have little effect on patient outcomes if therapists are providing guideline adherent care at baseline. Other authors [49] suggest that the duration of the implementation strategy may be insufficient to produce long-term changes in practice and patient outcomes. Additionally, adherence was measured inconsistently between studies in this review. Defining and utilizing a singular assessment may provide better insight into provider adherence.

Providers report numerous barriers to guideline adherence including patient preference [50]. However, most CPGs now recommend patient-centered care and shared decision-making (SDM) as an important component of care, thus placing greater emphasis on patient preference [51]. Guidance for implementation of SDM has been identified as an area that is lacking [52] and was typically not assessed as a component of adherence in the included studies. Improving the application of SDM in guideline adherent care may improve patient outcomes and satisfaction with treatment.

In addition to the above, it is worth considering the source of the guidelines. The quality of the evidence the guidelines are created from may impact outcomes and adherence [53]. In this review, we included studies assessing implementation of any published guideline. Therefore, there may be a difference in quality and outcomes based on the guidelines assessed.

Finally, though improving adherence to guidelines through implementation strategies may improve quality of care in neck and low back pain, a change in patient outcomes may be difficult to assess. This could be due to the generally favorable outcomes of acute low back [54] and neck pain [55]. Therefore, using HCU as a proxy assessment of quality of care may be appropriate. As noted above, delineating chronicity of pain may also be of use in future studies.

### Healthcare utilization

Similar to our findings, a systematic review by Hanney et al. [56] found that guideline adherence reduced HCU in patients treated for LBP. Our systematic review added additional data from thirteen new studies. These studies provided further support for the benefit of reducing HCU when adhering to guidelines. Moreover, our systematic review also captures findings from studies that have investigated the impact of guideline adherent care for neck pain. Furthermore, new studies included in our review included the assessment of guideline-based care in conjunction with additional treatments.

A plausible reason for the reduction in healthcare costs, healthcare visits, and resource utilization may be in part because many guidelines recommend fewer visits (typically three or less) for acute low back pain, instead promoting independence through reassurance and advice to remain active [17, 21]. Thus, guideline adherent care would trend toward fewer physical therapy visits and subsequently lower costs, as demonstrated in this review. However, reduction in physical therapy visits alone does not account for the reduction of non-physical therapy-related HCU.

### Pain and function

Pain and function improved in all groups in the included studies. Of the included studies assessing pain, only two found significant improvements favoring guideline implementation. The lack of improvement in patient-reported function and pain following guideline implementation is consistent with findings in other reviews [37, 49, 57]. Some authors [29] have suggested this lack of improvement may be due to high-quality care already provided by physical therapists. Others [37] have stated this may be due to additional patient characteristics, such as fear-avoidance behaviors that limit change. This may be supported by one study [26] which compared guideline-based care plus pain education to guideline-based care alone and found greater improvements in pain in the intervention group. Emerging evidence suggests that there may be a benefit of including new treatment approaches within guideline adherent care.

Of note, several studies assessed impacts in relation to chronicity of LBP. Two studies [17, 18] found greater improvements in acute low back pain with guideline adherence, whereas one study [35] found a significant effect on pain in patients with chronic low back pain. This suggests that subgroup analyses for chronicity of low back pain should be considered in future studies.

In our review, five studies [17, 18, 27, 33, 35] found a significant difference in patient-reported functional outcomes favoring guideline adherent care. Of these, only one study [33] compared implementation to a no-implementation control group and found no significant difference in functional outcomes. The remaining studies did not compare implementation strategies or have a control group. Therefore, it is difficult to determine the efficacy of implementation strategies on functional outcomes.

The ability to compare functional outcomes across studies is limited due to the lack of consistent outcome measures utilized. It is important to note that the majority of the studies that found improvements favoring guideline adherence used the ODI as the primary functional outcome measure. A recent review indicated that most patient-reported outcome measures assessing physical functioning in low back pain were insufficient to garner a full understanding of the patient’s physical functioning [58]. Another study recently found that the RMDQ and ODI cannot be used interchangeably [59]. Determining a valid and reliable physical functioning measure to utilize across studies will aid in determining effectiveness of implementation.

### Neck pain

Only two of the 21 included studies assessed guidelines associated with neck pain. One study assessed chronic neck pain [19], and one assessed WAD [28]. One study [19] found that the group that received nonadherent care had greater improvements in pain, but no difference in functional outcomes. More research is needed to assess the impact of guideline implementation and adherence on patient outcomes in patients with neck pain.

### Future research

The results of this review suggest guideline implementation and adherence may decrease HCU, but the results are inconclusive when comparing pain and physical function outcomes. However, as only one study used a no intervention comparator, the true effect of the implementation strategies is unknown and should be further investigated. Due to the limited studies identified, future research should also focus on implementation of guidelines in neck pain.

### Strengths

This systematic review is the first to our knowledge that assesses the impact of physical therapist implementation of CPGs on patient-level outcomes in back and neck pain. Our search strategy was thorough and able to capture as many eligible studies as possible. We utilized Powell et al. [14] to classify implementation strategies as we found this to be the most comprehensive, incorporating the elements of the Effective Practice and Organization of Care (EPOC) system, a checklist for systematic reviews. The EPOC system requires many studies to be excluded based on study design. Therefore, by utilizing the Powell et al. classification system, we were able to include a greater number of studies.

### Limitations

Several limitations to this review exist. There was significant heterogeneity across studies related to the type of implementation, the interventions used, and measured outcomes, limiting the ability to effectively synthesize the results. We utilized a broad definition of implementation during our inclusion process to better capture current practice. However, it is unlikely that a more narrow definition would have significantly reduced the heterogeneity encountered.

Because we utilized the classification system proposed by Powell et al. [14], additional studies qualified for inclusion compared to similar, previous studies. Key implementation processes and implementation strategies were assigned based on reviewer interpretation of included study articles and therefore some nuances of implementation may have been missed. We excluded studies that assessed effectiveness of implementation via vignettes as we did not find this to be an accurate representation of clinical practice [60]. Additionally, this review only assessed patient-level outcomes and did not assess impact on use of guideline-based care.

## Conclusion

In conclusion, guideline implementation in physical therapy treatment of low back and neck pain has a positive effect on HCU, but more research is needed to determine the effect on pain and function. This may be further elucidated by analyzing chronicity of pain separately. Additionally, utilizing a uniform outcome measure to assess function may further highlight the effect of implementation, or lack thereof. The most effective implementation strategy is unknown, but use of blended or published implementation frameworks may help guide effective strategies. However, the reduction in HCU, without sacrificing pain and functional outcomes, improves the value of care provided and demonstrates the benefits of guideline implementation.

## Supplementary Information

**Additional file 1.** Search Strategy Report.

## Data Availability

Not applicable.
