# Supplementary material for: Does the implementation of clinical practice guidelines for low back and neck pain by physical therapists improve patient outcomes? A systematic review
Source: Implement Sci Commun. 2022 Jun 3;3:57. doi: 10.1186/s43058-022-00305-2 (PMC9164354; doi:10.1186/s43058-022-00305-2)
Supplement: Supplementary file 1 — Additional file 1. Search Strategy Report. [file 43058_2022_305_MOESM1_ESM.docx]

**Additional file 1: Search Strategy Report:**

Steph Hendren, MLIS; Duke University Medical Center Library, Duke University School of Medicine

Date of completed original search: 4.28.2020

Total number of citations from original search: 1509

Date of updated search: 5.21.2020

Total number of citations from updated search: 268

Database: MEDLINE (via Ovid)

| 1. | exp Physical therapy Modalities/ OR exp Physical Therapists/ OR exp Physical therapy Specialty/ OR exp Rehabilitation/ OR exp Physical Therapist Assistants/ OR rehabilitation.fs. OR ("physical therapy" OR "physical therapist" OR "physical therapists" OR physiotherapy OR physiotherapies OR physiotherapist OR physiotherapists OR rehabilitation OR rehabilitating OR rehabilitated).ti,ab. | 536249 |
| --- | --- | --- |
| 2. | exp Back Pain/ OR exp sciatica/ OR exp Neck Pain/ OR (backache OR backaches OR coccydynia OR cervicalgia).ti,ab. | 49024 |
| 3. | exp spine/ OR exp back/ OR exp "Spinal Diseases"/ OR exp Coccyx/ OR exp Sacrum/ OR exp Spinal Canal/ OR exp Neck/ OR (back OR backs OR neck OR necks OR lumbar OR spine OR spinal OR vertebra OR vertebrae OR vertebral OR vertebrogenic OR intervertebral OR disc OR discs OR disk OR disks OR sacral OR sacrum OR tailbone OR "tail bone" OR coccyx OR lumbosacral OR lumbopelvic OR sciatic OR spondylosis OR spondylolysis OR spondylolisthesis).ti,ab. | 984085 |
| 4. | exp pain/ OR exp "pain management"/ OR exp "pain perception"/ OR (pain OR pains OR painful OR pained OR ache OR aches OR aching OR discomfort OR discomforts OR cramp OR cramps OR strain OR strains OR strained OR stiff OR stiffness OR sciatica OR lumbago OR nociception OR algesia).ti,ab. | 1615704 |
| 5. | exp Guideline Adherence/ OR ((exp prevalence/ OR (prevalence OR rate OR rates OR nonadherence OR non-adherence OR "non adherence" OR adherence OR adhering OR adhered OR adherent OR implementing OR implementation OR implementations OR implemented OR implement OR compliance OR compliant).ti,ab.) AND (exp Guideline/ OR exp Practice Guidelines as Topic/ OR (guidelines OR guideline OR CPG OR CPGs OR "best practice" OR "best practices").ti,ab.)) | 155192 |
| 6. | 2 OR (3 AND 4) | 183891 |
| 7. | 1 AND 5 AND 6 | 365 |

Updated Search: 5.21.2021

| 1. | exp Physical therapy Modalities/ OR exp Physical Therapists/ OR exp Physical therapy Specialty/ OR exp Rehabilitation/ OR exp Physical Therapist Assistants/ OR rehabilitation.fs. OR ("physical therapy" OR "physical therapist" OR "physical therapists" OR physiotherapy OR physiotherapies OR physiotherapist OR physiotherapists OR rehabilitation OR rehabilitating OR rehabilitated).ti,ab. | 567983 |
| --- | --- | --- |
| 2. | exp Back Pain/ OR exp sciatica/ OR exp Neck Pain/ OR (backache OR backaches OR coccydynia OR cervicalgia).ti,ab. | 51646 |
| 3. | exp spine/ OR exp back/ OR exp "Spinal Diseases"/ OR exp Coccyx/ OR exp Sacrum/ OR exp Spinal Canal/ OR exp Neck/ OR (back OR backs OR neck OR necks OR lumbar OR spine OR spinal OR vertebra OR vertebrae OR vertebral OR vertebrogenic OR intervertebral OR disc OR discs OR disk OR disks OR sacral OR sacrum OR tailbone OR "tail bone" OR coccyx OR lumbosacral OR lumbopelvic OR sciatic OR spondylosis OR spondylolysis OR spondylolisthesis).ti,ab. | 1043452 |
| 4. | exp pain/ OR exp "pain management"/ OR exp "pain perception"/ OR (pain OR pains OR painful OR pained OR ache OR aches OR aching OR discomfort OR discomforts OR cramp OR cramps OR strain OR strains OR strained OR stiff OR stiffness OR sciatica OR lumbago OR nociception OR algesia).ti,ab. | 1721467 |
| 5. | exp Guideline Adherence/ OR ((exp prevalence/ OR (prevalence OR rate OR rates OR nonadherence OR non-adherence OR "non adherence" OR adherence OR adhering OR adhered OR adherent OR implementing OR implementation OR implementations OR implemented OR implement OR compliance OR compliant).ti,ab.) AND (exp Guideline/ OR exp Practice Guidelines as Topic/ OR (guidelines OR guideline OR CPG OR CPGs OR "best practice" OR "best practices").ti,ab.)) | 174237 |
| 6. | 2 OR (3 AND 4) | 196807 |
| 7. | 1 AND 5 AND 6 | 423 |
|  | Publication date: 2020 - present | 72 |

Database: Embase (via Elsevier)

Note: search conducted in "results" tab

| 1. | 'physiotherapy'/exp OR 'physiotherapist'/exp OR 'rehabilitation'/exp OR **'rehabilitation':lnk OR** ('physical therapy' OR 'physical therapist' OR 'physical therapists' OR physiotherapy OR physiotherapies OR physiotherapist OR physiotherapists OR rehabilitation OR rehabilitating OR rehabilitated):ab,ti | 692171 |
| --- | --- | --- |
| 2. | 'backache'/exp OR 'sciatica'/exp OR 'neck pain'/exp OR (backache OR backaches OR coccydynia OR cervicalgia):ab,ti | 132291 |
| 3. | 'spine'/exp OR 'back'/exp OR 'spine disease'/exp OR 'coccyx'/exp OR 'sacrum'/exp OR 'neck'/exp OR (back OR backs OR neck OR necks OR lumbar OR spine OR spinal OR vertebra OR vertebrae OR vertebral OR vertebrogenic OR intervertebral OR disc OR discs OR disk OR disks OR sacral OR sacrum OR tailbone OR 'tail bone' OR coccyx OR lumbosacral OR lumbopelvic OR sciatic OR spondylosis OR spondylolysis OR spondylolisthesis):ab,ti | 1428698 |
| 4. | 'pain'/exp OR 'analgesia'/exp OR 'nociception'/exp OR (pain OR pains OR painful OR pained OR ache OR aches OR aching OR discomfort OR discomforts OR cramp OR cramps OR strain OR strains OR strained OR stiff OR stiffness OR sciatica OR lumbago OR nociception OR algesia):ab,ti | 2698618 |
| 5. | 'protocol compliance'/exp OR ('prevalence'/exp OR (prevalence OR rate OR rates OR nonadherence OR non-adherence OR 'non adherence' OR adherence OR adhering OR adhered OR adherent OR implementing OR implementation OR implementations OR implemented OR implement OR compliance OR compliant):ab,ti AND ('practice guideline'/exp OR (guidelines OR guideline OR CPG OR CPGs OR 'best practice' OR 'best practices'):ab,ti)) | 287854 |
| 6. | 2 OR (3 AND 4) | 355295 |
| 7. | 1 AND 5 AND 6 | 713 |

Updated Search: 5.21.2021

| 1. | 'physiotherapy'/exp OR 'physiotherapist'/exp OR 'rehabilitation'/exp OR **'rehabilitation':lnk OR** ('physical therapy' OR 'physical therapist' OR 'physical therapists' OR physiotherapy OR physiotherapies OR physiotherapist OR physiotherapists OR rehabilitation OR rehabilitating OR rehabilitated):ab,ti | 743536 |
| --- | --- | --- |
| 2. | 'backache'/exp OR 'sciatica'/exp OR 'neck pain'/exp OR (backache OR backaches OR coccydynia OR cervicalgia):ab,ti | 143137 |
| 3. | 'spine'/exp OR 'back'/exp OR 'spine disease'/exp OR 'coccyx'/exp OR 'sacrum'/exp OR 'neck'/exp OR (back OR backs OR neck OR necks OR lumbar OR spine OR spinal OR vertebra OR vertebrae OR vertebral OR vertebrogenic OR intervertebral OR disc OR discs OR disk OR disks OR sacral OR sacrum OR tailbone OR 'tail bone' OR coccyx OR lumbosacral OR lumbopelvic OR sciatic OR spondylosis OR spondylolysis OR spondylolisthesis):ab,ti | 1522565 |
| 4. | 'pain'/exp OR 'analgesia'/exp OR 'nociception'/exp OR (pain OR pains OR painful OR pained OR ache OR aches OR aching OR discomfort OR discomforts OR cramp OR cramps OR strain OR strains OR strained OR stiff OR stiffness OR sciatica OR lumbago OR nociception OR algesia):ab,ti | 2888871 |
| 5. | 'protocol compliance'/exp OR ('prevalence'/exp OR (prevalence OR rate OR rates OR nonadherence OR non-adherence OR 'non adherence' OR adherence OR adhering OR adhered OR adherent OR implementing OR implementation OR implementations OR implemented OR implement OR compliance OR compliant):ab,ti AND ('practice guideline'/exp OR (guidelines OR guideline OR CPG OR CPGs OR 'best practice' OR 'best practices'):ab,ti)) | 325163 |
| 6. | 2 OR (3 AND 4) | 383629 |
| 7. | 1 AND 5 AND 6 | 825 |
|  | Publication Dates: 2020 - present | 121 |

Database: CINAHL Complete (via EbscoHost)

| 1. | MH "Rehabilitation+" OR MH "Physical Therapy+" OR MH "Physical Therapy Practice, Evidence-Based" OR MW "rh" OR TI ("physical therapy" OR "physical therapist" OR "physical therapists" OR physiotherapy OR physiotherapies OR physiotherapist OR physiotherapists OR rehabilitation OR rehabilitating) OR AB("physical therapy" OR "physical therapist" OR "physical therapists" OR physiotherapy OR physiotherapies OR physiotherapist OR physiotherapists OR rehabilitation OR rehabilitating OR rehabilitated) | 415701 |
| --- | --- | --- |
| 2. | MH "Sciatica" OR MH "Back Pain+" OR MH "Neck Pain+" OR TI (backache OR bachaches OR coccydynia OR cervicalgia) OR AB (backache OR backaches OR coccydynia OR cervicalgia) | 39431 |
| 3. | MH "Spine+" OR MH "Back" OR MH "Lumbar Vertebrae" OR MH "Intervertebral Disk+" OR MH "Spinal Diseases+" OR MH "Neck" OR TI (back OR backs OR neck OR necks OR lumbar OR spine OR spinal OR vertebra OR vertebrae OR vertebral OR vertebrogenic OR intervertebral OR disc OR discs OR disk OR disks OR sacral OR sacrum OR tailbone OR "tail bone" OR coccyx OR lumbosacral OR lumbopelvic OR sciatic OR spondylosis OR spondylolysis OR spondylolisthesis) OR AB (back OR backs OR neck OR necks OR lumbar OR spine OR spinal OR vertebra OR vertebrae OR vertebral OR vertebrogenic OR intervertebral OR disc OR discs OR disk OR disks OR sacral OR sacrum OR tailbone OR "tail bone" OR coccyx OR lumbosacral OR lumbopelvic OR sciatic OR spondylosis OR spondylolysis OR spondylolisthesis) | 254540 |
| 4. | MH "Pain+" OR MH "Pain Management" OR TI (pain OR pains OR painful OR pained OR ache OR aches OR aching OR discomfort OR discomforts OR cramp OR cramps OR strain OR strains OR strained OR stiff OR stiffness OR sciatica OR lumbago OR nociception OR algesia) OR AB (pain OR pains OR painful OR pained OR ache OR aches OR aching OR discomfort OR discomforts OR cramp OR cramps OR strain OR strains OR strained OR stiff OR stiffness OR sciatica OR lumbago OR nociception OR algesia) | 409794 |
| 5. | MH "Guideline Adherence" OR ((TI (nonadherence OR non-adherence OR "non adherence" OR adherence OR adhering OR adhered OR adherent OR implementing OR implementation OR implementations OR implemented OR implement OR compliance OR compliant) OR AB (nonadherence OR non-adherence OR "non adherence" OR adherence OR adhering OR adhered OR adherent OR implementing OR implementation OR implementations OR implemented OR implement OR compliance OR compliant)) AND (MH "Practice Guidelines" OR TI (guidelines OR guideline OR CPG OR CPGs OR "best practice" OR "best practices") OR AB (guidelines OR guideline OR CPG OR CPGs OR "best practice" OR "best practices"))) | 46809 |
| 6. | 2 OR (3 AND 4) | 72235 |
| 7. | 1 AND 5 AND 6 | 187 |

Updated Search: 5.21.2021

| 1. | MH "Rehabilitation+" OR MH "Physical Therapy+" OR MH "Physical Therapy Practice, Evidence-Based" OR MW "rh" OR TI ("physical therapy" OR "physical therapist" OR "physical therapists" OR physiotherapy OR physiotherapies OR physiotherapist OR physiotherapists OR rehabilitation OR rehabilitating) OR AB("physical therapy" OR "physical therapist" OR "physical therapists" OR physiotherapy OR physiotherapies OR physiotherapist OR physiotherapists OR rehabilitation OR rehabilitating OR rehabilitated) | 411294 |
| --- | --- | --- |
| 2. | MH "Sciatica" OR MH "Back Pain+" OR MH "Neck Pain+" OR TI (backache OR bachaches OR coccydynia OR cervicalgia) OR AB (backache OR backaches OR coccydynia OR cervicalgia) | 39052 |
| 3. | MH "Spine+" OR MH "Back" OR MH "Lumbar Vertebrae" OR MH "Intervertebral Disk+" OR MH "Spinal Diseases+" OR MH "Neck" OR TI (back OR backs OR neck OR necks OR lumbar OR spine OR spinal OR vertebra OR vertebrae OR vertebral OR vertebrogenic OR intervertebral OR disc OR discs OR disk OR disks OR sacral OR sacrum OR tailbone OR "tail bone" OR coccyx OR lumbosacral OR lumbopelvic OR sciatic OR spondylosis OR spondylolysis OR spondylolisthesis) OR AB (back OR backs OR neck OR necks OR lumbar OR spine OR spinal OR vertebra OR vertebrae OR vertebral OR vertebrogenic OR intervertebral OR disc OR discs OR disk OR disks OR sacral OR sacrum OR tailbone OR "tail bone" OR coccyx OR lumbosacral OR lumbopelvic OR sciatic OR spondylosis OR spondylolysis OR spondylolisthesis) | 257826 |
| 4. | MH "Pain+" OR MH "Pain Management" OR TI (pain OR pains OR painful OR pained OR ache OR aches OR aching OR discomfort OR discomforts OR cramp OR cramps OR strain OR strains OR strained OR stiff OR stiffness OR sciatica OR lumbago OR nociception OR algesia) OR AB (pain OR pains OR painful OR pained OR ache OR aches OR aching OR discomfort OR discomforts OR cramp OR cramps OR strain OR strains OR strained OR stiff OR stiffness OR sciatica OR lumbago OR nociception OR algesia) | 412365 |
| 5. | MH "Guideline Adherence" OR ((TI (nonadherence OR non-adherence OR "non adherence" OR adherence OR adhering OR adhered OR adherent OR implementing OR implementation OR implementations OR implemented OR implement OR compliance OR compliant) OR AB (nonadherence OR non-adherence OR "non adherence" OR adherence OR adhering OR adhered OR adherent OR implementing OR implementation OR implementations OR implemented OR implement OR compliance OR compliant)) AND (MH "Practice Guidelines" OR TI (guidelines OR guideline OR CPG OR CPGs OR "best practice" OR "best practices") OR AB (guidelines OR guideline OR CPG OR CPGs OR "best practice" OR "best practices"))) | 47369 |
| 6. | 2 OR (3 AND 4) | 79771 |
| 7. | 1 AND 5 AND 6 | 200 |
|  | Publication dates: 2020 - present | 20 |

Database: Scopus (via Elsevier)

| 1. | TITLE-ABS({physical therapy} OR {physical therapist} OR {physical therapists} OR physiotherapy OR physiotherapies OR physiotherapist OR physiotherapists OR rehabilitation OR rehabilitating OR rehabilitated) | 322325 |
| --- | --- | --- |
| 2. | TITLE-ABS(backache OR backaches OR coccydynia OR cervicalgia) | 3557 |
| 3. | TITLE-ABS(back OR backs OR neck OR necks OR lumbar OR spine OR spinal OR vertebra OR vertebrae OR vertebral OR vertebrogenic OR intervertebral OR disc OR discs OR disk OR disks OR sacral OR sacrum OR tailbone OR {tail bone} OR coccyx OR lumbosacral OR lumbopelvic OR sciatic OR spondylosis OR spondylolysis OR spondylolisthesis) | 1794005 |
| 4. | TITLE-ABS(pain OR pains OR painful OR pained OR ache OR aches OR aching OR discomfort OR discomforts OR cramp OR cramps OR strain OR strains OR strained OR stiff OR stiffness OR sciatica OR lumbago OR nociception OR algesia) | 2615809 |
| 5. | TITLE-ABS( guidelines OR guideline OR CPG OR CPGs OR {best practice} OR {best practices}) | 684069 |
| 6. | TITLE-ABS(prevalence OR rate OR rates OR nonadherence OR non-adherence OR {non adherence} OR adherence OR adhering OR adhered OR adherent OR implementing OR implementation OR implementations OR implemented OR implement OR compliance OR compliant) | 9538324 |
| 7. | 2 OR (3 AND 4) | 216908 |
| 8. | 1 AND 5 AND 6 AND 7 | 244 |

Updated Search: 5.21.2021

| 1. | TITLE-ABS({physical therapy} OR {physical therapist} OR {physical therapists} OR physiotherapy OR physiotherapies OR physiotherapist OR physiotherapists OR rehabilitation OR rehabilitating OR rehabilitated) | 340529 |
| --- | --- | --- |
| 2. | TITLE-ABS(backache OR backaches OR coccydynia OR cervicalgia) | 3705 |
| 3. | TITLE-ABS(back OR backs OR neck OR necks OR lumbar OR spine OR spinal OR vertebra OR vertebrae OR vertebral OR vertebrogenic OR intervertebral OR disc OR discs OR disk OR disks OR sacral OR sacrum OR tailbone OR {tail bone} OR coccyx OR lumbosacral OR lumbopelvic OR sciatic OR spondylosis OR spondylolysis OR spondylolisthesis) | 1901798 |
| 4. | TITLE-ABS(pain OR pains OR painful OR pained OR ache OR aches OR aching OR discomfort OR discomforts OR cramp OR cramps OR strain OR strains OR strained OR stiff OR stiffness OR sciatica OR lumbago OR nociception OR algesia) | 2793755 |
| 5. | TITLE-ABS( guidelines OR guideline OR CPG OR CPGs OR {best practice} OR {best practices}) | 756790 |
| 6. | TITLE-ABS(prevalence OR rate OR rates OR nonadherence OR non-adherence OR {non adherence} OR adherence OR adhering OR adhered OR adherent OR implementing OR implementation OR implementations OR implemented OR implement OR compliance OR compliant) | 10296796 |
| 7. | 2 OR (3 AND 4) | 232678 |
| 8. | 1 AND 5 AND 6 AND 7 | 283 |
|  | Publication Dates: 2020 - present | 55 |
